# Exome-Wide Analysis Identifies a Rare *EXD3* Missense Variant Associated With Diabetic Kidney Disease

**DOI:** 10.1016/j.ekir.2025.09.053

**Published:** 2025-10-22

**Authors:** Niina Sandholm, Joanne B. Cole, Viji Nair, Eoin Brennan, Elena Giardini, Jani K. Haukka, Eunji Ha, Anna Syreeni, Emma H. Dahlström, Rany M. Salem, Damian Fermin, Josep Mercader, Laura Smyth, Claire Hill, Josyf Mychaleckyj, Stuart McGurnaghan, Rachel G. Miller, Tina Costacou, Barbara E.K. Klein, Janet Snell-Bergeon, Andrew D. Paterson, Rasa Verkauskiene, Jelizaveta Sokolovska, Nicolae Mircea Panduru, Gianpaolo Zerbini, Kerstin Brismar, Andrzej S. Krolewski, Valma Harjutsalo, Peter Rossing, Samy Hadjadj, Gareth McKay, Amy Jayne McKnight, Alexander P. Maxwell, Katalin Susztak, Catherine Godson, Matthias Kretzler, Joel N. Hirschhorn, Jose C. Florez, Per-Henrik Groop, GENIE Consortium, Joel N. Hirschhorn, Jose C. Florez, Xiaoqi Luo, Emma H. Dahlström, Anna Syreeni, Erkka Valo, Valma Harjutsalo, Per-Henrik Groop, Niina Sandholm, Laura J. Smyth, Katie Kerr, Jill Kilner, Yogesh Gupta, Claire Hill, Christopher Wooster, Kerry Anderson, Amy Jayne McKnight, Alexander P. Maxwell, Ciarán Kennedy, Elena Giardini, Ross Doyle, Eoin Brennan, Darrell Andrews, Denise Sadlier, Finian Martin, Catherine Godson, Viji Nair, Damian Fermin, Lalita Subramanian, Matthias Kretzler, Hongbo Liu, Katalin Susztak, Rany M. Salem, Joanne B. Cole

**Affiliations:** 1 Folkhälsan Research Center, Helsinki, Finland; 2 Department of Nephrology, University of Helsinki and Helsinki University Hospital, Helsinki, Finland; 3 Research Program for Clinical and Molecular Metabolism, Faculty of Medicine, University of Helsinki, Helsinki, Finland; 4 Programs in Metabolism and Medical and Population Genetics, Broad Institute of Harvard and MIT, Cambridge, Massachusetts, USA; 5 Center for Genomic Medicine, Massachusetts General Hospital, Boston, Massachusetts, USA; 6 Department of Medicine, Harvard Medical School, Boston, Massachusetts, USA; 7 Division of Endocrinology, Boston Children’s Hospital, Boston, Massachusetts, USA; 8 Department of Biomedical Informatics, University of Colorado School of Medicine, Aurora, Colorado, USA; 9 Department of Medicine-Nephrology, University of Michigan School of Medicine, Ann Arbor, Michigan, USA; 10 Diabetes Complications Research Centre, Conway Institute, School of Medicine, University College Dublin, Dublin, Ireland; 11 Renal Electrolyte and Hypertension Division, Department of Medicine, University of Pennsylvania, Philadelphia, Pennsylvania, USA; 12 Institute of Diabetes Obesity and Metabolism, University of Pennsylvania, Philadelphia, Pennsylvania, USA; 13 Department of Genetics, University of Pennsylvania, Philadelphia, Pennsylvania, USA; 14 Herbert Wertheim School of Public Health and Human Longevity Science, University of California San Diego, La Jolla, California, USA; 15 Department of Pediatrics-Nephrology, University of Michigan School of Medicine, Ann Arbor, Michigan, USA; 16 Diabetes Unit, Endocrine Division, Department of Medicine, Massachusetts General Hospital, Boston, Massachusetts, USA; 17 Centre for Public Health, Queen’s University of Belfast, Belfast, UK; 18 Center for Public Health Genomics, University of Virginia, Charlottesville, Virginia, USA; 19 The Institute of Genetics and Cancer, University of Edinburgh, Western General Hospital, Edinburgh, UK; 20 Department of Epidemiology, School of Public Health, University of Pittsburgh, Pittsburgh, Pennsylvania, USA; 21 Department of Ophthalmology and Visual Sciences, University of Wisconsin School of Medicine and Public Health, Madison Wisconsin, USA; 22 Barbara Davis Center for Diabetes, University of Colorado Denver, Aurora [CACTI], Colorado, USA; 23 Genetics and Genome Biology Research Institute, SickKids Hospital, Toronto, Ontario, Canada; 24 Institute of Endocrinology, Lithuanian University of Health Sciences, Kaunas, Lithuania; 25 Faculty of Medicine and Life Sciences, University of Latvia, Riga, Latvia; 26 2nd Clinical Department, Carol Davila University of Medicine and Pharmacy, Bucharest, Romania; 27 IMI – Center of Diabetes, Nutrition and Metabolism, Bucharest, Romania; 28 Complications of Diabetes Unit, Division of Immunology, Transplantation and Infectious Diseases, Diabetes Research Institute, IRCCS San Raffaele Scientific Institute, Milano, Italy; 29 Department of Molecular Medicine and Surgery, Rolf Luft Center for Diabetes Research and Endocrinology, Karolinska Institutet, Stockholm, Sweden; 30 Department of Endocrinology, Diabetes and Metabolism, Karolinska University Hospital, Stockholm, Sweden; 31 Section on Genetics and Epidemiology, Research Division, Joslin Diabetes Center, Boston, Massachusetts, USA; 32 Steno Diabetes Center Copenhagen, Herlev, Denmark; 33 Department of Clinical Medicine, University of Copenhagen, Copenhagen, Denmark; 34 CIC 1402 and U 1082, INSERM (National Institute of Health and Medical Research), Poitiers, France; 35 Department of Endocrinology, L'institut du thorax, INSERM, CNRS, Centre Hospitalier Universitaire de Nantes, Nantes, France; 36 Regional Nephrology Unit, Belfast City Hospital, Belfast, UK; 37 Penn-CHOP Kidney Innovation Center, University of Pennsylvania, Philadelphia, Pennsylvania, USA; 38 Department of Internal Medicine, University of Michigan, Ann Arbor, Michigan, USA; 39 Department of Pediatrics and Genetics, Harvard Medical School, Boston, Massachusetts, USA; 40 Department of Diabetes, Central Clinical School, Monash University, Melbourne, Victoria, Australia; 41 Baker Heart and Diabetes Institute, Melbourne, Victoria, Australia

**Keywords:** diabetic kidney disease, *EXD3*, exome analysis, missense variants, MUC5B, type 1 diabetes

## Abstract

**Introduction:**

Diabetic kidney disease (DKD) is a major complication of diabetes, with genetic factors contributing to its progression. Although genome-wide association studies (GWAS) have identified common variants, the role of low-frequency and rare coding variants remains underexplored.

**Methods:**

We performed exome-wide meta-analysis of up to 10,312 individuals with type 1 diabetes (T1D) genotyped using genome arrays with focused exome content. We included 10 DKD definitions based on albuminuria, estimated glomerular filtration rate (eGFR), or both. We analyzed nonsynonymous variants individually and used gene-level analyses for low-frequency (minor allele frequency [MAF] < 5%) and rare (< 1%) variants. Replication was performed in 10,066 participants with T1D and in UK Biobank participants with type 2 diabetes (T2D). Gene expression was assessed in cultured human podocytes.

**Results:**

In addition to the known *COL4A3* variant, a novel rare missense variant in *EXD3* (p.Asp555Asn, rs200080727, minor allele frequency [MAF] = 0.4%) was associated with DKD (odds ratio [OR] = 8.7, *P* = 4.5 × 10^-9^). The variant was predicted to be deleterious and *EXD3* was downregulated in DKD in kidney expression datasets. *EXD3* knock-down in a cultured human podocyte cell line reduced nephrin gene expression, suggesting a functional role in podocyte biology. Gene-level analyses identified 7 DKD-associated genes (*P* < 3.4 × 10^−6^), including *MUC5B*, which harbored multiple low-frequency missense variants and with evidence of replication. Replication in UK Biobank supported the association of *EXD3* rs200080727 with albuminuria (*P* = 0.014).

**Conclusion:**

This study identified a rare *EXD3* variant with a strong effect on DKD risk in T1D. Functional data support a role for *EXD3* in podocyte integrity and DKD pathogenesis. However, further functional investigations are necessary to understand the underlying molecular mechanisms.

## Graphical abstract

**Figure:**
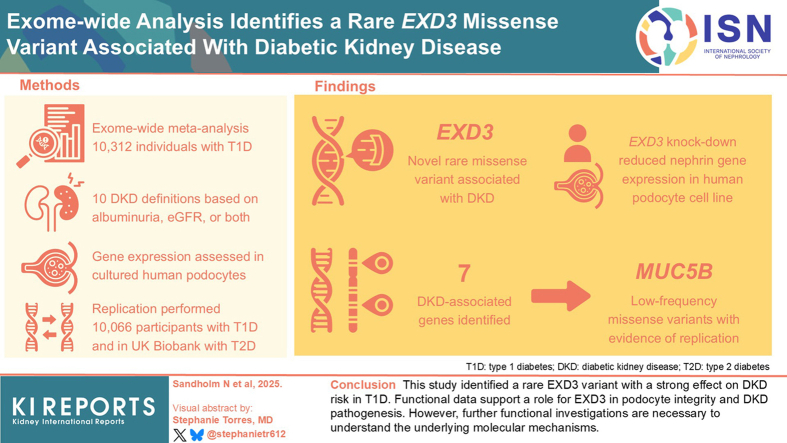

With > 500 million individuals affected globally, diabetes is the leading cause of chronic kidney disease (CKD). Despite improvements in diabetes care, nearly half of individuals with T1D and severe albuminuria still progress to kidney failure within 20 years.^1^ Although hyperglycemia is the main risk factor for DKD, genetic factors further exacerbate DKD risk.^2^ GWAS have identified tens of variants associated with DKD or other kidney-related traits in individuals with diabetes.^3^, ^4^, ^5^, ^6^, ^7^, ^8^, ^9^, ^10^, ^11^, ^12^ The largest GWAS on DKD in T1D, including nearly 20,000 individuals, identified 16 genetic loci. Although GWAS findings for complex diseases are enriched for regulatory variants,^13^ the locus with the strongest evidence of association for DKD was a common missense variant in the *COL4A3* gene encoding type IV collagen α3 chain, a major structural component of the glomerular basement membrane.^7^ Importantly, rare *COL4A3* variants cause Alport syndrome, characterized by kidney disease. Furthermore, many lead variants in the same GWAS were low-frequency (MAF < 5%) or rare (MAF < 1%).^7^

Although common variants have the greatest population-level impact on common diseases, low-frequency and rare variants may have a higher impact on the individual level.^14^ For example, 71% of *LDLR* mutation carriers had hypercholesterolemia in the UK Biobank whole-exome sequencing data,^15^ motivating the search for low-frequency missense variants for DKD as well. A previous GWAS meta-analysis focusing on rare variants identified protective missense variants in the *HSD17B14* gene associated with DKD.^16^ Furthermore, a genome sequencing study of 76 sibling pairs with T1D suggested involvement of protein kinases in DKD,^17^ and an exome and genome sequencing study including 1064 unrelated individuals with T1D identified a missense variant in the *LTA* gene, encoding for tumor necrosis factor-β, associated with lower tumor necrosis factor receptor levels and reduced risk of DKD.^18^ However, these sequencing studies suffer from limited statistical power because of modest sample sizes. Exome arrays provide a cost-effective alternative, capturing > 80% of the low-frequency and rare coding variants (MAF: 0.01%−5%) in Europeans,^19^ enabling analysis in larger cohorts. Here, we performed exome-wide analysis of GWAS studies on DKD, genotyped with arrays covering both common variant and exome array content, with the aim to further study the role of low-frequency and rare coding variants in DKD (Figure 1).

**Figure 1:**
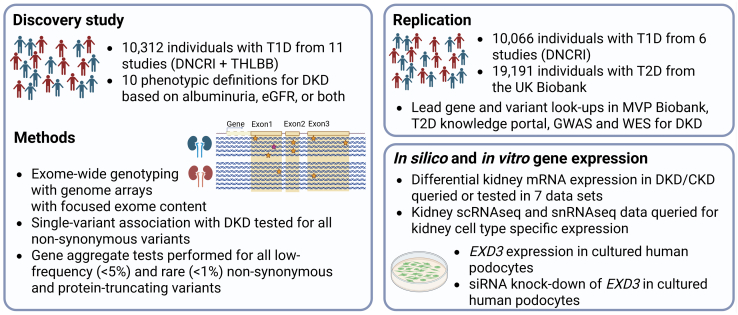
Study overview. CKD, chronic kidney disease; DKD, diabetic kidney disease; DNCRI, Diabetic Nephropathy Collaborative Research Initiative.^7^; GWAS, genome-wide association study; mRNA, messenger RNA; scRNAseq, single-cell RNA sequencing; siRNA, small interfering RNA; snRNAseq, single-nucleus RNA sequencing; T1D, type 1 diabetes; T2D, type 2 diabetes; THLBB, Finnish THL Biobank; MVP, Million Veteran Program; WES, whole-exome sequencing. Created in BioRender.

## Methods

### Study Design

The discovery study included up to 10,312 individuals with T1D and of European origin from 11 cohorts (Figure 1, Supplementary Table S1). Ten of the cohorts were part of a previous GWAS meta-analysis, centrally genotyped with the Illumina HumanCoreExome arrays^7^; in addition, we included GWAS data for 2356 individuals with T1D from the Finnish THL Biobank. We performed both single variant analysis and gene aggregate tests to identify missense variants and genes associated with DKD. Replication of the lead variants was assessed using GWAS summary statistics for DKD from up to 10,066 participants with T1D, and further evidence of replication was assessed in the UK Biobank individuals with diabetes.^20^

### Participants

All participants had T1D as diagnosed by their attending physician, with age at diabetes onset ≤ 40 years. DKD was assessed based on either albuminuria (classified as normal [albumin excretion rate, AER: < 20 μg/min or equivalent], moderate [AER: 20–200 μg/min or equivalent] or severe albuminuria [AER: ≥ 200 μg/min or equivalent] or kidney failure), eGFR, or both. Ten phenotype definitions were based on the various thresholds for disease severity as described previously (Supplementary Table S2).^7^

Ten cohorts were genotyped at the University of Virginia with the Illumina HumanCoreExome chip. Variant calling, data quality control, and genotype imputation with the 1000 Genomes Project or Finnish SISU v3 sequencing data as the reference have been described earlier^7^^,^^21^ and are detailed in the Supplementary Methods. In addition, we obtained imputed genotype data for the THL Biobank T1D samples.

### Statistical Analysis

Statistical analysis is described in detail in the Supplementary Methods. Briefly, association analysis was performed in each cohort for nonsynonymous variants with high imputation quality (*r*^*2*^ ≥ 0.95) using rvtests score test. Analyses were adjusted for age, genetic sex, diabetes duration, study-specific covariates (e.g., study center or genotyping batch), and either the top principal components or a kinship matrix. Variant covariance matrices were calculated in 500 k base pairs sliding windows.

Single variant and gene aggregate test meta-analyses were performed with raremetal (https://genome.sph.umich.edu/wiki/RAREMETAL). All encountered variants were pooled and annotated with ANNO variant annotation tool. Single variant meta-analysis was performed using an inverse-variance approach, and the results were filtered to variants present in ≥ 2 studies and with total minor allele count ≥ 5 across studies. Exome-wide significance was defined as *P*-value < 5 × 10^−7^.

To increase statistical power to detect genes with coding variants associated with DKD, we used 3 types of gene aggregate tests to collectively analyze the variants within each gene. These tests included a burden test that collapsed and combined all valid variants with a given MAF threshold, a variable threshold burden test that defined the optimal MAF cut-off, and a SKAT kernel-based aggregate test that allows variants to have either a protective or risk-increasing effect and is more robust to the inclusion of benign variants. Gene aggregate analyses were performed separately for any nonsynonymous variants, including missense and nonsense variants; and for the more severe protein-truncating variants including nonsense and nonstop variants, start loss or gain, frameshift, or essential splice site variants. Variants were filtered to those with MAF ≤ 5%, ≤ 1%, or < 0.5%. Gene aggregate test meta-analyses were performed with raremetal based on the single variant score test summary statistics and variant covariance matrices. Cohort-wise summary statistics were filtered to variants with minor allele count ≥ 3 before meta-analysis. *Post hoc* analyses were repeated without the study-wise filtering of minor allele count ≥ 3.

Gene aggregate results were filtered to those with ≥ 2 variants. *P*-values < 3.4 × 10^−6^ (corrected for 14,963 genes with nonsynonymous variants) and < 2.5 × 10^-5^ (corrected for 2013 genes with protein-truncating variants) were considered significant for burden of nonsynonymous and protein-truncating variants, respectively.

The lead variants were annotated with Ensembl b37 variant effect predictor for the most severe consequence, including the Combined Annotation Dependent Depletion score (e.g., Combined Annotation Dependent Depletion score > 20 indicates top 1% of most damaging variants^22^) and for SIFT, PolyPhen2 predictions of the variant effect.

### Replication

We attempted single variant replication of all nonsynonymous variants found in *EXD3* and the 7 significant genes. Replication was tested in a meta-analysis of previously calculated summary statistics from DNCRI studies where individual level data was not available and therefore not included in the gene aggregate analyses (Supplementary Table S1). Variants were filtered to those with imputation INFO ≥ 0.9 and annotated with Ensemble variant effect predictor as nonsynonymous or protein-truncating variants; 186 variants were identified within the 8 genes. *P*-values < 2.7 × 10^−4^, that is, corrected for 186 variants, were considered significant.

Replication for *EXD3* rs200080727 was tested in *n* = 19,191 unrelated UK Biobank participants with T2D (application number 27892) with the *EXD3* p.Asp555Asn minor allele count of 158 (MAF: 0.4%). CKD-DKD status was determined using the most recent albumin-to-creatinine ratio and eGFR. Association was additionally tested for albumin-to-creatinine ratio and eGFR as continuous traits (Supplementary Methods).

We performed association look-ups for all nonsynonymous variants within the 8 lead genes in a GWAS meta-analysis for DKD in T2D,^4^ and in a whole-exome sequencing for DKD, including 4372 individuals with and without diabetes in their discovery stage analysis,^20^ with results available for variants with *P* < 1 × 10^−5^. Finally, we queried the lead variants in the VA Million Veteran Program phenome-wide association study results of 2068 traits derived from electronic health records for 635,969 participants^23^ in the CIPHER Million Veteran Program gwPheWAS PheWeb portal (https://phenomics.va.ornl.gov/pheweb/gia/meta/, accessed February 19, 2025); and the lead genes for kidney-related gene-level rare variant associations in the T2D Knowledge portal (https://t2d.hugeamp.org/, accessed February 2, 2025).

### *EXD3* rs200080727 Genotype Validation

The *EXD3* rs200080727 variant was directly genotyped on HumanCoreExome Bead array 12-1.0 in the first data freeze, but not on later versions 12-1.1 and 24-1.0 used to genotype subsequent cohorts.^7^ In FinnDiane, genotyping occurred in 4 batches across all 3 arrays, whereby only variants available on all arrays were included in the final merged genotype file. We extracted FinnDiane genotypes from the first batch, genotyped with HumanCoreExome Bead array 12-1.0, with genotyping rate > 99.9% and 14 rs200080727 variant carriers detected among 5013 samples (MAF: 0.14%). Whole-exome sequencing and whole-genome sequencing data were available for 2354 participants (similar to 1064 samples as previously described),^18^ of which 1996 had overlapping GWAS data, used to calculate the concordance of the rs200080727 genotype calls between the genotyping chip and sequencing data.

### Differential Gene Expression Analysis

Differential gene expression was evaluated with limma regression^24^ in European Renal cDNA Bank microarray data for microdissected glomerular (*n* = 12; GSE104948-glom) and tubular (*n* = 17; GSE104954-tub) samples from individuals with a histological diagnosis of diabetic nephropathy versus *n* = 48 glomerular or *n* = 46 tubular samples from living donors. Six of the 8 queried genes were detected.^25^

*EXD3* and *MUC5B* kidney gene expressions were queried in the Nephroseq database (http://v5.nephroseq.org) in the Nakagawa CKD Kidney,^26^ Woroniecka Diabetes,^27^ Schmid Diabetes,^28^ and Ju CKD^25^ datasets. In addition, they were studied in 2 additional RNA-sequencing analyses of DKD from Fan *et al.*^29^ and Levin *et al.*,^30^ with mRNA read counts available as described earlier.^31^ Levin *et al.*^30^ provided raw read counts; therefore, genes with ≤ 2 counts in ≥ 31 of 39 samples were filtered out, and trimmed mean of M normalization was applied. Fan *et al.* provided normalized read counts,^29^ so no further processing was needed. Differential expression was analyzed on the Galaxy platform (Galaxy Version 3.48.0+galaxy1, www.usegalaxy.org)^32^ using the limma-voom method^33^ with default options. *P*-values were adjusted using the Benjamini and Hochberg method.^34^

*EXD3* and *MUC5B* gene expression in kidney single-cell and single-nucleus RNA sequencing data were queried in the Humphreys Lab portal^35^ (http://humphreyslab.com/SingleCell/, Accessed 01/24/2025) and at the Susztaklab Kidney Biobank portal (http://www.susztaklab.com/, Accessed 20/1/2025) based on kidney single-cell transcriptome atlas of > 200,000 cells from human normal and disease kidneys (merged Susztak & KPMP data).^36^

### Human Podocyte Cell Line

A conditionally immortalized human podocyte cell line was kindly provided by Dr Richard Coward and Professor Moin Saleem (Bristol University).^37^ The cells were cultured in RPMI 1640 (Gibco -31870-074) supplemented with 10%(v/v) heat-inactivated fetal bovine serum, 100× ITS (Sigma I3146), L-glutamine (2 mM) and penicillin/streptomycin (100 U/ml). Cells were grown to 60% confluency at 33 °C before temperature switching to 37 °C and differentiating for 14 days. For treatments, differentiated cells were stimulated with tumor necrosis factor-α (10 ng/ml), platelet-derived growth factor (10ng/ml), TGFβ (10 ng/ml), high glucose (25 mM) and nephrotoxin puromycin aminonucleoside (PAN, 25 μg/ml) for 24 hours as indicated. Cells were transfected for 24 hours with *EXD3* small interfering RNA pools or control small interfering RNA (20 nmol/l; 24 hours) according to the manufacturer's instructions using Lipofectamine RNAimax (Thermo Fisher). RNA was extracted using the E.N.Z.A. TOTAL RNA KIT 1 (Omega Bio-Tek), and cDNA synthesis was performed using Superscript II reverse transcriptase, following the manufacturer’s recommendation. Gene expression analysis was performed using TaqMan reagents (Life Technologies) and normalized to GAPDH rRNA using the ΔΔCt method.^38^

## Results

### Single Variant Meta-Analysis

**Figure 2:**
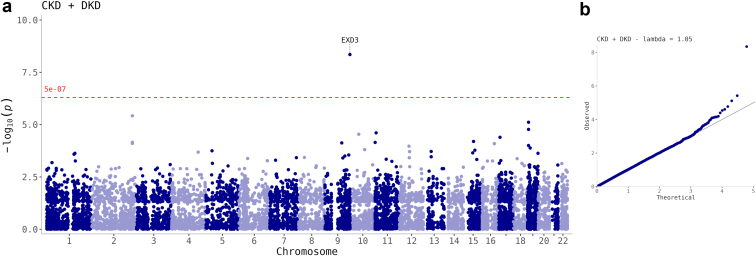
Single-variant association analysis discovered an *EXD3* variant associated with the CKD + DKD phenotype. (a) Manhattan plot of the association. The horizontal dashed line indicates exome-wide significance, i.e., *P*-value < 5 × 10^−7^. (b) QQ-plot of association. CKD + DKD: cases with albuminuria (albumin excretion rate, albumin excretion rate ≥ 20 μg/min or equivalent) and estimated glomerular filtration rate < 45 ml/min per 1.73 m^2^, controls with normal albumin excretion rate and eGFR ≥ 60 ml/min per 1.73 m^2^. CKD, chronic kidney disease; DKD, diabetic kidney disease.

In single variant meta-analysis, we had 80% power to detect a low-frequency variant (MAF: ≤ 5%) with an OR ≥ 1.47; or a rare variant (MAF: ≤ 1%) with OR ≥ 2.32 (*α =* 5 × 10^−7^; Supplementary Figure S1). In addition to our previously identified common *COL4A3* p.Asp326Tyr variant (rs55703767; *P* = 5.0 × 10^−8^), a novel, rare (MAF: 0.4%) *EXD3* p.Asp555Asn variant (rs200080727 C/T, *P* = 4.5 × 10^−^^9^) was associated with the combined CKD + DKD phenotype, that is, cases with albuminuria (AER ≥ 20 μg/min or equivalent) and eGFR < 45 ml/min per 1.73 m^2^ (Table 1, Figure 2, Supplementary Figures S2 and S3). By pooling the genotype counts across the contributing studies, the variant was estimated to have a crude OR of 8.7 (Supplementary Table S3). In addition, *EXD3* p.Asp555Asn was associated with other DKD phenotype definitions, albeit below the exome-wide significance threshold (Supplementary Table S4). Carriers of the rs200080727 T allele had lower average eGFR (36.3 vs. 81.0 ml/min per 1.73 m^2^, *P* = 0.009) and higher diastolic blood pressure (90 vs. 78, *P* = 0.004; Supplementary Table S5). The variant was predicted to be deleterious, probably damaging, and likely pathogenic by SIFT, Polyphen2, and AlphaMissense^39^ algorithms, with a Combined Annotation Dependent Depletion score > 20, that is, among 1% of most damaging variants, suggesting that the DKD-associated allele may result in a loss of gene function. Furthermore, 3 other missense variants in the *EXD3* gene were nominally associated (*P* < 0.05) with the CKD + DKD phenotype, and 2 of them were predicted to be deleterious by the SIFT algorithm (Supplementary Table S6).

**Table 1:** Single non-synonymous variants associated with DKD (*P* < 5 × 10^−7^)

Phenotype	Chr:pos	rsID	REF	ALT	*n*	ALT AF	Direction	Beta	P-value	gene	Conseq	SIFT	PolyPhen2
CKD + DKD	9:140,243,729	rs20008072	C	T	1086	0.004	??????++−	8.10	4.5 × 10^−9^	*EXD3*	p.Asp555Asn	0: deleterious	1: probably damaging
Severe DKD	2:228,121,101	rs55703767	G	T	10,000	0.20	−−−−−−−+−−	−0.23	5.0 × 10^−8^	*COL4A3*	p.Asp326Tyr	0.06: tolerated	0.157: benign

Phenotype: CKD + DKD: cases with albuminuria (AER ≥ 20 μg/min or equivalent) and eGFR < 45 ml/min per 1.73 m^2^, controls with normal albumin excretion rate and eGFR ≥ 60 ml/min per 1.73 m^2^. Severe DKD: cases with severe albuminuria (AER ≥ 200 μg/min or equivalent) or kidney failure, controls with normal AER. Chr:pos: chromosome and base pair position (GRCh37). The 3 last studies with +/- effects for rs200080727 are Italy, Steno, and the Sweden cohorts (Supplementary Table S2). rs200080727 is directly genotyped on the Illumina HumanCoreExome Bead array 12-1.0; rs55703767 is directly genotyped on all used HumanCoreExome Bead arrays 12-1.0, 12-1.1 and 24-1.0. AER, albumin excretion rate; ALT AF, alternative allele frequency; ALT, alternative allele; Beta, effect size estimate; CKD, chronic kidney disease; Conseq, amino acid consequence for the primary transcript annotated with Ensembl variant effect predictor; Direction: direction of effect (for the ALT allele) in each contributing study: +: increased risk, −: decreased risk, ?: not available/detected; DKD, diabetic kidney disease; eGFR, estimated glomerular filtration rate; *n*: number of samples; Polyphen2, polyphen2 score and interpretation, obtained with Ensembl b37 variant effect predictor; REF, reference allele; SIFT, SIFT score and interpretation.

### Replication and Validation of *EXD3* p.Asp555Asn Variant (rs200080727)

No carriers of the *EXD3* p.Asp555Asn variant (rs200080727) were detected in the T1D replication cohorts. However, extending the analysis to other nonsynonymous *EXD3* variants identified 2 missense variants associated with CKD + DKD (*P* < 0.05; Supplementary Table S6). Furthermore, the variant was detected in the first genotyping batch of one of the discovery cohorts (FinnDiane, *n* = 5013, genotyping rate > 99.9%) and confirmed by sequencing (100% concordance in 1996 overlapping samples), although it was not associated with the CKD + DKD phenotype (Supplementary Table S7). Replication in the UK Biobank individuals with T2D indicated that rs200080727 was associated with albuminuria in diabetes (P = 0.014, Supplementary Table S8). Further look-ups from external databases showed that rs200080727 was associated with dialysis in the Million Veteran Program (*P* = 0.002, OR = 2.0); rare variants in *EXD3* were associated with CKD in the T2D Knowledge portal (*P* = 7.6 × 10^−4^ for “LOF + missense 0.5 (MAF < 0.001%)” filter, OR = 2.53, *n* = 132,351). Finally, rs200080727 is located 140 k base pairs away from rs28404308, a common variant GWAS locus for eGFR in general population.^40^

### *EXD3* Gene Expression in Kidneys

We queried *EXD3* gene expression in multiple kidney gene expression datasets. *EXD3* gene was significantly underexpressed in kidneys in individuals with CKD compared with healthy controls^26^ (fold change: −2.59, *P* = 5.0 × 10^−14^; Supplementary Table S9). Kidney single-nucleus RNA sequencing indicated enriched *EXD3* expression in kidney podocytes (Supplementary Figure S4), with some evidence for *EXD3* expression in other cell types, including injured proximal tubules (Supplementary Table S10).

**Figure 3:**
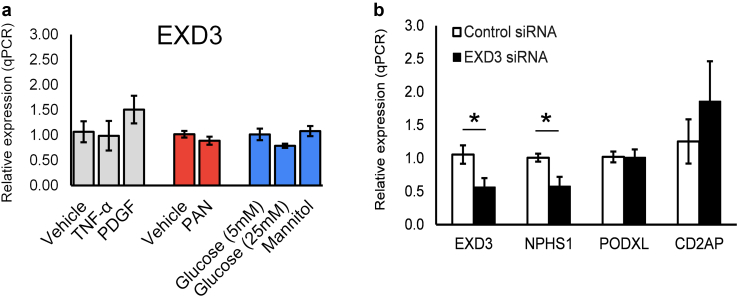
*EXD3* in podocyte biology. (a) Cultured conditionally immortalized human podocytes (hPODs) were differentiated for 14 days at 37 °C and treated with TNF -α (10 ng/ml), PDGF (10 ng/ml) and nephrotoxin PAN (25 μg/ml) for 24 hours. For glucose stimulations, hPODs were stimulated with low glucose (5 mM), high glucose (25 mM) or mannitol (20 mM) for 24 hours. (b) hPODs were transfected with *EXD3* siRNA pools or control siRNA (20 nmol/l; 24 hours) for 24 hours. Gene expression analysis of *EXD3, NPHS1, PODXL*, and *CD2AP* was performed using TaqMan reagents (Life Technologies) and normalized to GAPDH rRNA using the ΔΔCt method. Data are presented as *n* = 3 ± SEM. ∗*t* test *P* < 0.05. PAN, puromycin aminonucleoside; PDGF, platelet-derived growth factor; siRNA, small interfering RNA; TNF--α, tumor necrosis factor-α.

We next studied the role of *EDX3* in podocytes using conditionally immortalized cultured human podocytes. Of note, *EXD3* expression was unchanged in response to stimuli, including tumor necrosis factor-α, platelet-derived growth factor, and nephrotoxin PAN. Furthermore, *EXD3* expression did not change by glucose stimulations (Figure 3a). To mimic the observation of reduced *EXD3* expression in human CKD kidney tissue, we performed small interfering RNA–mediated knockdown of *EXD3* human podocytes to test the hypothesis that reduced *EXD3* levels in podocytes leads to cell dysfunction. Of interest, knock-down of *EXD3* was associated with a significant reduction in nephrin (*NPHS1)* expression (Figure 3b), providing evidence of a putative biological role for *EXD3* in podocyte biology.

### Seven DKD-Associated Genes Identified With Gene Aggregate Analyses

**Figure 4:**
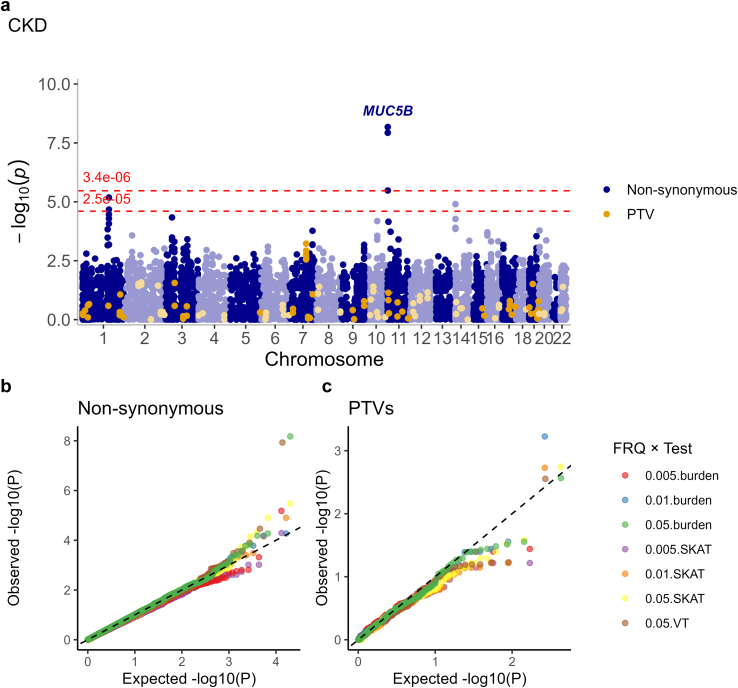
Gene aggregate analysis identified missense variants in the *MUC5B* gene associated with the “CKD” phenotype definition in T1D. (a) Manhattan plot of gene aggregate test results for the “CKD” phenotype definition, that is, eGFR < 60 ml/min per 1.73 m^2^. Gene aggregate test results for nonsynonymous variants are drawn with purple color, significant association defined as *P*-value < 3.4 × 10^−6^ (upper dashed red line, corrected for 14,963 genes with nonsynonymous variants). Results for PTVs are drawn with orange color, significant associations defined as *P*-values < 2.5 × 10^−5^ (lower dashed red line, corrected for 2013 genes with protein-truncating variants). (b) Quantile-quantile plot (QQ-plot) for gene aggregate tests of nonsynonymous variants, stratified by aggregate test (burden, SKAT, or variable threshold (VT)) and variant frequency. (c) QQ-plot for gene aggregate tests of protein-truncating variants, stratified by aggregate test and variant frequency. Manhattan and QQ-plots for other diabetic kidney disease phenotype definitions are provided in Supplementary Figure S5. CKD, chronic kidney disease; PTV, protein-truncating variants.

We performed gene aggregate tests to increase the statistical power to identify genes with multiple nonsynonymous variants, or more severe protein-truncating variants, associated with DKD (*P* < 3.4 × 10^−6^; Supplementary Figure S5). The strongest association was found for missense variants in *IGSF3* (Ig superfamily member 3) associated with the kidney failure versus no kidney failure (*P* = 1.5 × 10^−10^; Table 2, Figure 4). Furthermore, *IGSF3* was overexpressed in DKD kidneys (Supplementary Table S11). The genotype association was driven by a predicted deleterious p.Cys3Tyr variant (rs749817295 *P* = 2.5 × 10^−10^) but detected in only 1 study with a minor allele count of 6. Therefore, this finding needs further validation and must be interpreted with caution.

**Table 2:** Significant gene aggregate results (Bonferroni corrected *P* < 3.4 × 10^−6^ for nonsynonymous variants or *P* < 2.5 × 10^−5^ for protein-truncating variants)

Gene	Phenotype	Var types	Freq	*n* var	Cum mac	Test	*p*-value
*EOMES*	Any DKD	Missense	1%, 5%	2	224	SKAT	1.1 × 10^−6^
*IGSF3*	kidney failure vs. others	Missense	1%, 0.5%	2	11	SKAT	1.5 × 10^−10^
						Burden	2.8 × 10^−6^
*KIAA1109/ BLTP1*	Any DKD	Missense	5%	10	2409	SKAT	3.0 × 10^−6^
*LAIR1*	kidney failure vs. others	Missense	1%, 0.5%	3	20	SKAT	1.3 × 10^−6^
*MUC5B*	CKD	Missense	5%	65	13,161	Burden	6.7 × 10^−9^
						SKAT	3.3 × 10^−6^
			4.8%	63	11,551	VT	1.2 × 10^−8^
	CKD extremes	Missense	5%	63	11,368	Burden	1.6 × 10^−7^
			4.9%	61	9910	VT	1.3 × 10^−6^
*PLCB2*	kidney failure vs others	Missense	0.5%	4	47	Burden	2.2 × 10^−6^
			0.2%	3	44	VT	2.9 × 10^−6^
*ZAN*	CKD extremes	splice donor, frameshift, stop gained	1.9%	3	317	VT	3.4 × 10^−6^

Phenotype: Any DKD, cases with moderate or severe albuminuria (AER ≥ 20 μg/min or equivalent) or kidney failure, controls with normal AER. CKD: cases with eGFR < 60 ml/min per 1.73 m^2^, controls with eGFR ≥ 60 ml/min per 1.73 m^2^. CKD extremes: cases with eGFR < 15 ml/min per 1.73 m^2^ or kidney failure, controls with eGFR ≥ 90 ml/min per 1.73 m^2^. AER, albumin excretion rate; CKD, chronic kidney disease; Cum mac, cumulative minor allele count across all variants within the gene, across all studies; DKD, diabetic kidney disease; Freq: variant frequency threshold; *n* var, number of variants of the given variant type and below the given variant frequency threshold found within the gene; SKAT, sequence kernel association test; Test, gene aggregate test; Var types, variant types; VT, variable threshold burden test.

In contrast, *MUC5B* contained 65 missense variants with MAF ≤ 5% associated with the CKD phenotype (eGFR: ≤ 60 ml/min per 1.73 m^2^; *P* = 6.7 × 10^−9^). In addition, the gene was nominally associated (*P* < 0.05) with 5 other DKD definitions that included kidney failure in the case definition (Supplementary Table S12). Although the gene aggregate tests account for linkage disequilibrium through variant covariance matrices, 19 of 23 significantly associated variants (*P* < 0.05) were in high linkage disequilibrium with each other (*D’* = 1, *r*^*2*^ > 0.9; Supplementary Figure S6). The variants were in linkage disequilibrium with a synonymous variant rs2672810 (excluded from the current meta-analysis), which modestly tags length variation of a variable number tandem repeat (VNTR) in the large central exon (*r*^*2*^ = 0.66).^41^ However, rs2672810 was not associated with DKD in our previous GWAS.^7^ Kidney gene expression datasets resulted in inconclusive evidence of differential *MUC5B* kidney expression in DKD, with significant associations detected in both directions (Supplementary Table S9).

We tested replication of any nonsynonymous variants detected within the 7 significant genes from the gene aggregate analyses in the T1D replication cohorts. Four missense variants in *MUC5B* were nominally associated (*P* < 0.05) with the original DKD phenotype definition in which they were discovered; *MUC5B* p.Pro4357Leu (rs201463531) remained significant after correction for multiple testing (*P* = 9.4 × 10^−6^, minor allele count 4 in replication, not found in the discovery cohort; Supplementary Table S6).

In look-ups from external data, 2 missense variants in *MUC5B* were associated with DKD at the discovery stage of a whole-exome sequencing analysis (rs202127660/p.Asp682Gly *P* = 7.54 × 10^−11^ for DKD vs. CKD comparison, and rs1033142104/p.Val5715Gly *P* = 3.86 × 10^−5^ for DKD vs. healthy controls).^20^ Two further missense variants in *MUC5B*, p.Gly4862Ser/rs191989562 and p.Leu3193Pro/rs117913875, were associated (*P* < 0.05) with CKD in T2D.^4^

*Post hoc* gene aggregate analysis of the discovery data, including the rarest variants (minor allele count ≥ 1), identified 4 additional genes associated with DKD (*P* < 3.4 × 10^−6^; Supplementary Table S13), including *EMILIN1*, a kidney extracellular matrix protein that negatively regulates TGF-β signaling. These suggest involvement of very rare variants in DKD, but larger datasets are needed for robust validation.

## Discussion

We report findings from an exome-wide analysis of DKD in up to 10,312 individuals with T1D, based on reanalysis of GWAS data from 11 cohorts. This study revealed novel coding variants associated with DKD. In particular, a putative *EXD3* loss-of-function variant p.Asp555Asn (rs200080727) was associated with higher risk of DKD (OR = 8.7; *P* = 4.5 × 10^−9^), as well as the burden of 65 low frequency missense variants in the *MUC5B* gene (*P* = 6.7 × 10^−9^).

The *EXD3* p.Asp555Asn variant (rs200080727) was directly genotyped and detected in only 3 discovery cohorts. Although the findings did not replicate in the subset of FinnDiane study participants genotyped with the same array, its validity was supported by evidence of replication in UK Biobank individuals with T2D, the Million Veteran Program, and other genetic databases, as well as associations of other *EXD3* variants in T1D replication cohorts. The functional role of *EXD3* was supported by multiple gene expression datasets indicating lower *EXD3* expression in CKD or DKD, as well as in single-cell RNA sequencing and single-nucleus RNA sequencing data validating enriched *EXD3* expression in kidney podocytes. Finally, we demonstrated *EXD3* expression in a conditionally immortalized human podocyte cell line and showed that *EXD3* small interfering RNA silencing leads to reduced expression of nephrin, a key protein of the slit diaphragm. EXD3 and nephrin are both expressed in the podocytes, but their direct link is not well-characterized. EXD3 has putative 3'–5' exonuclease activity and is likely involved in RNA processing or DNA repair. However, EXD3 shows only broad nucleic acid binding prediction and is not validated for specific RNAs. It is plausible that EXD3 may regulate the levels of regulatory small RNAs, which in turn may regulate the levels of nephrin. However, further functional studies are needed to understand how reduced *EXD3* affects nephrin expression and the risk of DKD.

The burden of altogether 65 missense variants in *MUC5B* were associated with CKD in T1D, with evidence of replication both in individuals with T1D and T2D. *MUC5B* encodes a member of the mucin family, which are highly glycosylated macromolecular components of mucus secretions. Most mucins have a VNTR domain with great variation in the repeat number, which can alter mucin protein molecular weight even 2-fold.^42^ *MUC5B* is mainly expressed in saliva, lung mucus, cervical mucus, and to a lesser extent in normal kidneys (Supplementary Figure S4), with inconclusive evidence of differential *MUC5B* kidney expression in DKD. Interestingly, a *MUC5B* promoter variant rs35705950 is the strongest genetic risk factor for idiopathic pulmonary fibrosis.^43^ In addition, the promoter variant was associated with DKD in a look-up from our previous GWAS^7^ (*P* = 5.4 × 10^−4^, OR = 1.31). Thus, *MUC5B* variants may contribute to kidney fibrosis, a characteristic hallmark of DKD. Furthermore, many of the *MUC5B* missense variants associated with DKD were in linkage disequilibrium with a synonymous (nondetected) variant tagging the *MUC5B* VNTR length polymorphism; previously, *MUC5AC* VNTR length has been associated with cystic fibrosis lung disease.^44^ Of note, multiple single cytosine insertions in the coding VNTR region of *MUC1*, another mucin family member, were identified as the main cause of medullary cystic kidney disease type 1, an autosomal dominant tubulointerstitial kidney disease.^45^ Given the similar VNTR region in the *MUC5B* gene, it remains unclear whether the association with DKD is because of the observed missense variants, the tagged VNTR, or undetected frameshift variants similar to *MUC1*.

Despite the *in silico* functional annotation of *EXD3* rs200080727 as a probably damaging or deleterious variant, we note that this and the other DKD risk–associated variants reported in this paper would not meet the thresholds of "likely pathogenic" according to the American College of Medical Genetics criteria,^46^ but would be considered as variants of uncertain significance. In particular, we expect that the variants identified in our study do not represent fully penetrant monogenic forms of DKD, but instead risk increasing or decreasing variants with lower penetrance, even though with relatively higher ORs than expected for common variants. Whereas the American College of Medical Genetics classification framework was designed for highly penetrant monogenic diseases, the Clinical Genome Resource (ClinGen) Low Penetrance or Risk Allele Working Group suggests using risk alleles and low-penetrance variants as distinct variant classes.^47^ Following their classification, the missense variants identified in this study most closely match to the ”likely” or “uncertain risk allele” classification, depending on whether evidence of replication was found or not, with supporting functional evidence for the *EXD3* gene.

The main strengths of this study include the harmonized phenotype definitions of DKD, covering various degrees of disease severity, in line with our previous studies.^7^ Furthermore, including only individuals with T1D at the discovery stage ensures a more homogenous DKD phenotype than in individuals with T2D,^48^ more affected by other comorbidities such as aging, hypertension, and overweight. Ten of the 11 included cohorts were centrally genotyped with the Illumina HumanCoreExome chip, ensuring uniform data and processing of the rare variants. Furthermore, given the challenges in rare variant imputation,^49^ we included only imputed variants of high estimated quality (≥ 0.95) to avoid any bias related to rare variant imputation.

The gene aggregate analysis enabled us to detect genes with variants that were not sufficiently significant alone (e.g., *MUC5B* and *KIAA1109/BLTP1* missense variants) or reached significance with the more lenient *P*-value threshold for the gene tests compared with the single variant association tests (e.g. *LAIR1* and *EOMES*). Even though previous exome analyses of common diseases such as T2D have suggested a certain overlap of the exome and GWAS findings,^50^ we did not see overlap with previous GWAS findings for DKD, apart from the *COL4A3* common missense variant association.^7^ This might be because of the still limited number of genetic findings for DKD, supporting the use of alternative approaches such as the one reported here. Because a large proportion of missense variants are likely benign,^51^ filtering for likely pathogenic variants might further increase statistical power. Nevertheless, variants predicted as benign, such as the *COL4A3* p.Asp326Tyr (rs55703767), may be relevant, and gene aggregate methods such as SKAT^52^ are more robust to inclusion of benign variants.

Our exome array based study design allowed us to reach a sample number of > 10,000, which is an order of magnitude larger compared with the largest sequencing-based approaches for DKD to date (> 1000 participants^18^). The previous reports on similar exome arrays suggest that they can capture nearly 90% of low frequency and rare (MAF > 0.01%) variants in non-Finnish European populations^19^; because the MAF of 0.01% corresponds to 2 expected variant carriers within 10,000 samples, we expect the arrays to provide a good coverage of variants existing in our study cohorts. Nevertheless, we have likely missed many relevant rare variants, either not captured with the genotyping arrays or with genotype imputation, or because of limited statistical power to detect rare variant associations. Consequently, the sample size—both in the discovery and replication cohorts—remains the main limitation of the current study.

To conclude, our exome-wide analysis of DKD in > 10,000 participants with T1D identified rare missense variants associated with DKD. Supported by functional experiments and external data, these findings highlight *EXD3* as a potential target for future therapeutic interventions. However, further functional studies to clarify the precise role of the identified variants are warranted.

## Appendix

### List of the GENIE Consortium Members

Raymond Kreienkamp, Josep Mercader, Joel N. Hirschhorn, Jose C. Florez of the Massachusetts General Hospital and Broad Institute, Boston, Massachusetts, USA; Xiaoqi Luo, Emma H Dahlström, Anna Syreeni, Erkka Valo, Valma Harjutsalo, Per-Henrik Groop, and Niina Sandholm of The FinnDiane Study Group, Folkhälsan Research Center, University of Helsinki and Helsinki University Hospital, Helsinki, Finland; Laura J. Smyth, Katie Kerr, Jill Kilner, Yogesh Gupta, Claire Hill, Christopher Wooster, Kerry Anderson, Gareth J McKay, Amy Jayne McKnight, and Alexander P. Maxwell of Queen's University Belfast, Belfast, Northern Ireland; Ciarán Kennedy, Elena Giardini, Ross Doyle, Eoin Brennan, Darrell Andrews, Denise Sadlier, Finian Martin and Catherine Godson of the Diabetes Complications Research Centre, University College Dublin, Dublin, Ireland; Viji Nair, Damian Fermin, Lalita Subramanian, and Matthias Kretzler of University of Michigan School of Medicine, Ann Arbor, Michigan, USA; Eunji Ha, Hongbo Liu and Katalin Susztak of University of Pennsylvania, Perelman School of Medicine, Philadelphia, Pennsylvania, USA; Rany M Salem of University of California San Diego, La Jolla, California, USA; and Joanne B. Cole of University of Colorado School of Medicine, Aurora, Colorado, USA.

## Disclosure

RMS received funding from Travere Therapeutics. PR has received grants (to institution) from Novo Nordisk, AstraZeneca, and Bayer; and has received honoraria (to institution) from Abbott, AstraZeneca, Boehringer Ingelheim, Bayer, Eli Lilly, Novo Nordisk, Gilead, and Sanofi Aventis. MK reports grants and contracts outside the submitted work through the University of Michigan with NIH, Chan Zuckerberg Initiative, JDRF, AstraZeneca, Novo Nordisk, Eli Lilly, Gilead, Goldfinch Bio, Janssen, Boehringer-Ingelheim, Moderna, European Union Innovative Medicine Initiative, Certa, Chinook, amfAR, Angion, RenalytixAI, Travere, Regeneron, and IONIS; consulting fees through the University of Michigan from Astellas, Poxel, and Janssen; and a patent PCT/EP2014/073413 “Biomarkers and methods for progression prediction for chronic kidney disease” licensed. JNH holds equity in Camp4 Therapeutics. JCF has received consulting honoraria from Goldfinch Bio and AstraZeneca; and speaker fees from Novo Nordisk, AstraZeneca, and Merck for research lectures over which the author had full control of content. P-HG has received investigator-initiated research grants from Eli Lilly and Roche; is an advisory board member for AbbVie, Astellas, AstraZeneca, Bayer, Boehringer Ingelheim, Cebix, Eli Lilly, Janssen, Medscape, Merck Sharp & Dohme, Mundipharma, Nestlé, Novartis, Novo Nordisk, and Sanofi; and has received lecture fees from Astellas, AstraZeneca, Bayer, Berlin Chemie, Boehringer Ingelheim, Eli Lilly, Elo Water, Genzyme, Menarini, Merck Sharp & Dohme, Medscape, Mundipharma, Novartis, Novo Nordisk, PeerVoice, Sanofi, and Sciarc. All the other authors declared no competing interests.

## Supplementary Material

Supplementary File (PDF) **Supplementary Methods**. **Supplementary References**. **Figure S1**. Power calculation for severe DKD phenotype (*n* = 6177 controls with normal AER, *n* = 3823 cases with severe albuminuria or kidney failure), for α = 5 × 10^−7^ significance level. **Figure S2**. Single variant analysis Manhattan and QQ plots for each tested phenotype. **Figure S3**. *EXD3* p.Asp555Asn variant (rs200080727) association with CKD + DKD (*P* = 4.5 × 10^−9^). **Figure S4**. *EXD3* and *MUC5B* gene expression in single-nucleus RNA sequencing (snRNAseq) datasets. **Figure S5**. Manhattan and QQ-plots for the gene aggregate tests. **Figure S6**. Linkage disequilibrium structure between the *MUC5B* missense variants, the *MUC5B* promoter variant associated with gene expression (rs35705950), and rs2672810 tagging for a *MUC5B* VNTRS. **Table S1**. Number of cases and controls per phenotype definition. **Table S2**. DKD phenotype case and control definitions. **Table S3**. Genotype counts for *EXD3* rs200080727 across the 3 discovery cohorts. **Table S4**. *EXD3* p.Asp555Asn (rs200080727) association across the 10 phenotype definitions in the discovery cohort. **Table S5**. Clinical characteristics of the *EXD3* rs200080727 T allele carriers. **Table S6**. Lead gene nonsynonymous variants with *P*-value < 0.05 in discovery or replication stage. **Table S7**. *EXD3* p.Asp555Asn (rs200080727) association and genotype validation with sequencing data in the FinnDiane cohort subset. **Table S8**. *EXD3* p.Asp555Asn (rs200080727) replication in the UK Biobank individuals with type 2 diabetes. **Table S9**. *EXD3* and *MUC5B* gene expression in mRNA sequencing studies. **Table S10**. *EXD3* gene expression in single-nucleus (snRNA) and single-cell (scRNA) sequencing data of > 200,000 cells from human normal and disease kidneys. **Table S11**. Differential gene expression in the European Renal cDNA Bank microarray data for diabetic nephropathy versus living donors. **Table S12**. Lead gene associations across the 10 DKD definitions. **Table S13**. Gene aggregate results (*P* < 3.4 × 10^−6^ for nonsynonymous variants, *P* < 2.5 × 10^−5^ for protein-truncating variants) when also singletons and doubletons (i.e., minor allele count of 1 or 2) are included. **Table S14**. Physicians and nurses who contributed to patient recruitment and examination.
