# Supplementary material for: Exome-Wide Analysis Identifies a Rare EXD3 Missense Variant Associated With Diabetic Kidney Disease
Source: Kidney Int Rep. 2025 Oct 22;11(1):219–32. doi: 10.1016/j.ekir.2025.09.053 (PMC12799576; doi:10.1016/j.ekir.2025.09.053)
Supplement: Supplementary File (PDF) — Supplementary Methods. Supplementary References. Figure S1. Power calculation for severe DKD phenotype (n = 6177 controls with normal AER, n = 3823 cases with severe albuminuria or kidney failure), for α = 5 × 10^−7^ significance level. Figure S2. Single variant analysis Manhattan and QQ plots for each tested phenotype. Figure S3. EXD3 p.Asp555Asn variant (rs200080727) association with CKD + DKD (P = 4.5 × 10^−9^). Figure S4. EXD3 and MUC5B gene expression in single-nucleus RNA sequencing (snRNAseq) datasets. Figure S5. Manhattan and QQ-plots for the gene aggregate tests. Figure S6. Linkage disequilibrium structure between the MUC5B missense variants, the MUC5B promoter variant associated with gene expression (rs35705950), and rs2672810 tagging for a MUC5B VNTRS. Table S1. Number of cases and controls per phenotype definition. Table S2. DKD phenotype case and control definitions. Table S3. Genotype counts for EXD3 rs200080727 across the 3 discovery cohorts. Table S4. EXD3 p.Asp555Asn (rs200080727) association across the 10 phenotype definitions in the discovery cohort. Table S5. Clinical characteristics of the EXD3 rs200080727 T allele carriers. Table S6. Lead gene nonsynonymous variants with P-value < 0.05 in discovery or replication stage. Table S7. EXD3 p.Asp555Asn (rs200080727) association and genotype validation with sequencing data in the FinnDiane cohort subset. Table S8. EXD3 p.Asp555Asn (rs200080727) replication in the UK Biobank individuals with type 2 diabetes. Table S9. EXD3 and MUC5B gene expression in mRNA sequencing studies. Table S10. EXD3 gene expression in single-nucleus (snRNA) and single-cell (scRNA) sequencing data of > 200,000 cells from human normal and disease kidneys. Table S11. Differential gene expression in the European Renal cDNA Bank microarray data for diabetic nephropathy versus living donors. Table S12. Lead gene associations across the 10 DKD definitions. Table S13. Gene aggregate results (P < 3.4 × 10^−6^ for nonsynonymous variants, P < [file mmc1.pdf]

Supplementary Material

Exome-wide analysis identifies a rare *EXD3* missense variant associated with diabetic kidney disease

Niina Sandholm, Joanne B Cole, Viji Nair, Eoin Brennan, Elena Giardini, Jani K Haukka, Eunji Ha, Anna Syreeni, Emma H Dahlström, Rany M Salem, Damian Fermin, Josep Mercader, Laura Smyth, Claire Hill, Josyf Mychaleckyj, Stuart McGurnaghan, Rachel G Miller, Tina Costacou, Barbara E K Klein, Janet Snell-Bergeon, Andrew D Paterson, Rasa Verkauskiene, Jelizaveta Sokolovska, Nicolae Mircea Panduru, Gianpaolo Zerbini, Kerstin Brismar, Andrzej S Krolewski, Valma Harjutsalo, Peter Rossing, Samy Hadjadj, Gareth McKay, Amy Jayne McKnight, Alexander P Maxwell, Katalin Susztak, Catherine Godson, Matthias Kretzler, Joel N Hirschhorn, Jose C Florez, Per-Henrik Groop, GENIE Consortium

Contents

Supplementary Methods 3

GENIE Consortium – Full list of members 5

Supplementary Table S1: Number of cases and controls per phenotype definition 8

Supplementary Table S2: DKD phenotype case and control definitions 9

Supplementary table S3: Genotype counts for *EXD3* rs200080727 across the 3 discovery cohorts. 10

Supplementary Table S4: *EXD3* p.Asp555Asn (rs200080727) association across the 10 phenotype definitions in the discovery cohort. 11

Supplementary Table S5: Clinical characteristics of the *EXD3* rs200080727 T allele carriers 12

Supplementary Table S6: Lead gene non-synonymous variants with P-value <0.05 in discovery or replication stage 13

Supplementary Table S7: *EXD3* p.Asp555Asn (rs200080727) association and genotype validation with sequencing data in the FinnDiane cohort subset. 15

Supplementary Table S8: *EXD3* p.Asp555Asn (rs200080727) replication in the UK Biobank individuals with type 2 diabetes. 16

Supplementary Table S9: *EXD3* and *MUC5B* gene expression in mRNA sequencing studies. 17

Supplementary Table S10: *EXD3* gene expression in single nucleus (snRNA) and single cell (scRNA) sequencing data of >200,000 cells from human normal and disease kidneys. 19

Supplementary Table S11: Differential gene expression in the ERCB microarray data for diabetic nephropathy vs living donors. 22

Supplementary Table S12: Lead gene associations across the ten DKD definitions. 23

Supplementary Table S13: Gene aggregate results (p<3.4×10^{-6} for non-synonymous variants, p<2.5×10^{-5} for protein-truncating variants) when also singletons and doubletons (i.e., minor allele count of 1 or 2) are included. 25

Supplementary Table S14: Physicians and nurses contributing to the patient recruitment and examination 26

Supplementary Figure S1: Power calculation for Severe DKD phenotype (N=6,177 controls with normal AER, N=3,823 cases with severe albuminuria or kidney failure), for $\alpha=5\times10^{-7}$ significance level. 29

Supplementary Figure S2: Single variant analysis Manhattan and QQ-plots for each tested phenotype...... 30

Supplementary Figure S3: *EXD3* p.Asp555Asn variant (rs200080727) association with CKD+DKD ($p=4.5\times10^{-9}$). 33

Supplementary Figure S4: *EXD3* and *MUC5B* gene expression in single nucleus RNA sequencing (snRNAseq) datasets. Data queried from http://humphreyslab.com/SingleCell/ [S17] 34

Supplementary Figure S5: Manhattan and QQ-plots for the gene aggregate tests....................................... 35

Supplementary Figure S6: Linkage disequilibrium (LD) structure between the *MUC5B* missense variants, the *MUC5B* promoter variant associated with gene expression (rs35705950), and rs2672810 tagging for a *MUC5B* VNTR[S].. 38

Supplementary References 39

Supplementary Methods

Genotypic data

The study included 10,312 individuals of European origin with T1D from 11 cohorts. Ten of the cohorts were part of the GWAS meta-analysis for DKD as described previously[S1]; in addition, we included 2,356 Finnish individuals with T1D from the Finnish THL Biobank, after exclusion of individuals previously included as part of the Finnish Diabetic Nephropathy (FinnDiane) study.

Ten DNCRI cohorts were genotyped at the University of Virginia with the Illumina HumanCoreExome chip as previously described[S1]. Variants were identified using zCall software designed for calling rare variants. Variant quality control included filtering for low-quality variants (*e.g.*, call rates <95% and excessive deviation from Hardy–Weinberg equilibrium [HWE; $p < 1\times10^{-7}$ / $p < 1\times10^{-10}$ on HLA regions]) and samples (*e.g.*, call rates <98%, sex mismatch, extreme heterozygosity), and individuals with evidence of non-European ancestry (with principal component analysis) as described earlier.[S1] Non-Finnish samples were imputed to 1000 Genomes Project (phase 3v5) as the reference, as previously described.[S1] For FinnDiane, imputation was performed with a Finnish SISu v3 sequencing panel as the reference as described earlier.[S2]

We obtained imputed genotype data for the THL Biobank samples. Samples were genotyped as part of the FinnGen study at Thermo Fisher Scientific, San Diego, CA, USA, with the Affymetrix Axiom FinnGen1.r2 array. Genotype calls were made in FinnGen with AxiomGT1 algorithm, and variants were imputed with FinnGen R8 pipeline, imputed against the SISu v4 imputation reference panel.

Statistical analysis

Variants were filtered for those with high imputation quality ($r^2 \geq 0.95$). To limit the file size of the covariance matrices, the variants were annotated with SnpEff (https://pcingola.github.io/SnpEff/snpeff/introduction/) before analysis and filtered to any non-synonymous (exon loss variant, frameshift variant, stop gained, stop loss, start lost, splice acceptor variant, splice donor variant, missense variant, inframe insertion, disruptive inframe insertion, inframe deletion, or disruptive inframe deletions). Association analysis was performed in each cohort with rvtests score test meta-analysis model, which allows inclusion of related individuals. Analyses were adjusted for age, sex, diabetes duration, study specific covariates (e.g. study center or genotyping batch), and either the top principal components or a kinship matrix. Covariance matrices were simultaneously calculated for variants in sliding windows of 500 kbp.

Single variant and gene aggregate test meta-analyses were performed with raremetal based on the score test summary statistics and variant covariance matrices. All encountered variants across the studies were collated with raremetal (https://github.com/statgen/raremetal) and annotated with ANNO variant annotation tool (https://github.com/zhanxw/anno). Single variant meta-analysis was performed using an inverse-variance approach. Single variant meta-analysis results were filtered on the basis of their presence in at least two studies, and with cumulative minor allele count (CMAC) ≥5 across studies. Exome-wide significance was defined as *p*-value $<5\times10^{-7}$.

For gene aggregate meta-analysis, cohort-wise summary statistics were first filtered to variants with MAC ≥3 (i.e., excluding singletons and doubletons), and variants missing in a given cohort were not included in the meta-analysis (as opposed to setting them to 0 observations). Gene aggregate analyses included CMC type burden test that collapsed and combined all valid variants, a Variable Threshold (VT) burden test that defined the optimal MAF cut-off, and a SKAT kernel-based aggregate test that allowed variants to have either a protective or risk increasing effect. For SKAT analysis, p-value was calculated with the Liu method in raremetal. Gene aggregate analyses were performed separately for protein-truncating variants (start loss or gain, stop loss or gain, frameshift, or essential splice site variant) and any non-synonymous variants (non-synonymous, codon gain or loss variants, and protein-truncating variants), limiting variants to those with

minor allele frequency (MAF) ≤5 %, ≤1 % or <0.5 %. P-values < 3.4×10^{-6} (corrected for 14,963 genes with non-synonymous variants) and <2.5×10^{-5} (corrected for 2,013 genes with protein-truncating variants) were considered significant for burden of non-synonymous and protein-truncating variants, respectively. Gene aggregate results were filtered to those with at least 2 variants; with minimum MAC≥3 per variant, this resulted in minimum cumulative gene-wise CMAC of ≥ 6 across all studies. As sensitivity analysis, gene aggregate tests were repeated without the initial study-wise filtering of MAC ≥3.

The lead variants were annotated with Ensembl b37 Variant Effect Predictor (VEP) for the most severe consequence, including the CADD score (e.g., CADD score >20 indicates top 1% of most damaging variants[S3]) and for SIFT, PolyPhen2 predictions of the variant effect. In addition, we queried AlphaMissense[S4] prediction using the online portal at https://alphamissense.hegelab.org/search (accessed June 30, 2025).

Replication in the UK Biobank

Replication for *EXD3* rs200080727 was tested in the UK Biobank (application number 27892) participants with diabetes. Diabetes status in the UK Biobank was defined using a combination of a previously published algorithm[S5] and HbA1c levels. CKD-DKD status was determined using ACR and eGFR. ACR was calculated using urinary albumin (UKB field 30500) and urinary creatinine (UKB field 30510). eGFR was calculated using creatinine (UKB field 30700), the CKD Epi formula, and the nephro R package.[S6] The most recent measurements were used, and any individual urinary albumin measurement lower than the detection limit was set to the detection limit of 6.7 mg/L. Logistic and linear regressions on unrelated UK Biobank participants of European ancestry were adjusted for age, sex, diabetes duration, and genetic PCs. As only 30 individuals with type 1 diabetes were identified as CKD-DKD cases, we tested the association in the UK Biobank participants with type 2 diabetes (N=19,191), with EXD3p.Asp555Asn minor allele count (MAC) of 158 (MAF 0.4%).

GENIE Consortium – Full list of members

Name	Affiliations
**Massachusetts General Hospital and Broad Institute, Boston, MA, USA**	
Joel N Hirschhorn	Programs in Metabolism and Medical & Population Genetics, Broad Institute, Cambridge, MA USA.<br>Division of Endocrinology, Boston Children's Hospital, Boston, MA, USA<br>Department of Pediatrics and Genetics, Harvard Medical School, Boston, MA, USA
Jose C Florez	Programs in Metabolism and Medical & Population Genetics, Broad Institute, Cambridge, MA USA<br>Diabetes Unit and Center for Genomic Medicine, Massachusetts General Hospital, Boston, MA USA.<br>Department of Medicine, Harvard Medical School, Boston, MA USA.
Raymond Kreienkamp	Division of Endocrinology, Boston Children's Hospital, Boston, MA, USA<br>Diabetes Unit and Center for Genomic Medicine, Massachusetts General Hospital, Boston, MA USA.
**The FinnDiane Study Group, Folkhälsan Research Center, Helsinki, Finland**	
Xiaoqi Luo	Folkhälsan Institute of Genetics, Folkhälsan Research Center, Helsinki, Finland.<br>Department of Nephrology, University of Helsinki and Helsinki University Hospital, Helsinki, Finland.<br>Research Program for Clinical and Molecular Metabolism, Faculty of Medicine, University of Helsinki, 00290, Helsinki, Finland.
Emma H Dahlström	Folkhälsan Institute of Genetics, Folkhälsan Research Center, Helsinki, Finland.<br>Department of Nephrology, University of Helsinki and Helsinki University Hospital, Helsinki, Finland.<br>Research Program for Clinical and Molecular Metabolism, Faculty of Medicine, University of Helsinki, 00290, Helsinki, Finland.
Anna Syreeni	Folkhälsan Institute of Genetics, Folkhälsan Research Center, Helsinki, Finland.<br>Department of Nephrology, University of Helsinki and Helsinki University Hospital, Helsinki, Finland.<br>Research Program for Clinical and Molecular Metabolism, Faculty of Medicine, University of Helsinki, 00290, Helsinki, Finland.
Erkka Valo	Folkhälsan Institute of Genetics, Folkhälsan Research Center, Helsinki, Finland.<br>Department of Nephrology, University of Helsinki and Helsinki University Hospital, Helsinki, Finland.<br>Research Program for Clinical and Molecular Metabolism, Faculty of Medicine, University of Helsinki, 00290, Helsinki, Finland.
Valma Harjutsalo	Folkhälsan Institute of Genetics, Folkhälsan Research Center, Helsinki, Finland.<br>Department of Nephrology, University of Helsinki and Helsinki University Hospital, Helsinki, Finland.<br>Research Program for Clinical and Molecular Metabolism, Faculty of Medicine, University of Helsinki, 00290, Helsinki, Finland.
Per-Henrik Groop	Folkhälsan Institute of Genetics, Folkhälsan Research Center, Helsinki, Finland.<br>Department of Nephrology, University of Helsinki and Helsinki University Hospital, Helsinki, Finland.<br>Research Program for Clinical and Molecular Metabolism, Faculty of Medicine, University of Helsinki, 00290, Helsinki, Finland.

	Department of Diabetes, Central Clinical School, Monash University, Melbourne, Victoria, Australia.
Niina Sandholm	Folkhälsan Institute of Genetics, Folkhälsan Research Center, Helsinki, Finland.<br>Department of Nephrology, University of Helsinki and Helsinki University Hospital, Helsinki, Finland.<br>Research Program for Clinical and Molecular Metabolism, Faculty of Medicine, University of Helsinki, 00290, Helsinki, Finland.
**Queen's University Belfast, Belfast, Northern Ireland**	
Laura J Smyth	Molecular Epidemiology Research Group, Centre for Public Health, Queen's University Belfast, Belfast, UK.
Katie Kerr	Molecular Epidemiology Research Group, Centre for Public Health, Queen's University Belfast, Belfast, UK.
Jill Kilner	Molecular Epidemiology Research Group, Centre for Public Health, Queen's University Belfast, Belfast, UK.
Yogesh Gupta	Molecular Epidemiology Research Group, Centre for Public Health, Queen's University Belfast, Belfast, UK.
Claire Hill	Molecular Epidemiology Research Group, Centre for Public Health, Queen's University Belfast, Belfast, UK.
Christopher Wooster	Molecular Epidemiology Research Group, Centre for Public Health, Queen's University Belfast, Belfast, UK.
Kerry Anderson	Molecular Epidemiology Research Group, Centre for Public Health, Queen's University Belfast, Belfast, UK.
Gareth J McKay	Molecular Epidemiology Research Group, Centre for Public Health, Queen's University Belfast, Belfast, UK.
Amy Jayne McKnight	Molecular Epidemiology Research Group, Centre for Public Health, Queen's University Belfast, Belfast, UK.
Alexander P Maxwell	Molecular Epidemiology Research Group, Centre for Public Health, Queen's University Belfast, Belfast, UK.<br>Regional Nephrology Unit, Belfast City Hospital, Belfast, Northern Ireland, UK.
**Diabetes Complications Research Centre, University College Dublin, Dublin Ireland**	
Ciarán Kennedy	Diabetes Complications Research Centre, Conway Institute, School of Medicine, University College Dublin, Dublin Ireland.
Elena Giardini	Diabetes Complications Research Centre, Conway Institute, School of Medicine, University College Dublin, Dublin Ireland.
Ross Doyle	Diabetes Complications Research Centre, Conway Institute, School of Medicine, University College Dublin, Dublin Ireland.
Eoin Brennan	Diabetes Complications Research Centre, Conway Institute, School of Medicine, University College Dublin, Dublin Ireland.
Darrell Andrews	Diabetes Complications Research Centre, Conway Institute, School of Medicine, University College Dublin, Dublin Ireland.
Denise Sadlier	Mater Misericordiae Hospital, Dublin, Ireland D07 K201.
Finian Martin	Diabetes Complications Research Centre, Conway Institute, School of Medicine, University College Dublin, Dublin Ireland.

Catherine Godson	Diabetes Complications Research Centre, Conway Institute, School of Medicine, University College Dublin, Dublin Ireland.
**University of Michigan School of Medicine, Ann Arbor, MI, USA**	
Viji Nair	Department of Medicine-Nephrology, University of Michigan School of Medicine, Ann Arbor, MI 48109, USA.
Damian Fermin	Department of Pediatrics-Nephrology, University of Michigan School of Medicine, Ann Arbor, MI 48109, USA.
Lalita Subramanian	Department of Medicine-Nephrology, University of Michigan School of Medicine, Ann Arbor, MI 48109, USA.
Matthias Kretzler	Department of Internal Medicine, University of Michigan, Ann Arbor, Michigan, USA.
**University of Pennsylvania, Perelman School of Medicine, Philadelphia, PA, USA.**	
Hongbo Liu	Renal, Electrolyte, and Hypertension Division, Department of Medicine, University of Pennsylvania, Perelman School of Medicine, Philadelphia, PA, USA.<br>Institute for Diabetes, Obesity, and Metabolism, University of Pennsylvania, Perelman School of Medicine, Philadelphia, PA, USA.<br>Department of Genetics, University of Pennsylvania, Perelman School of Medicine, Philadelphia, PA, USA
Katalin Susztak	Renal, Electrolyte, and Hypertension Division, Department of Medicine, University of Pennsylvania, Perelman School of Medicine, Philadelphia, PA, USA.<br>Institute for Diabetes, Obesity, and Metabolism, University of Pennsylvania, Perelman School of Medicine, Philadelphia, PA, USA.<br>Department of Genetics, University of Pennsylvania, Perelman School of Medicine, Philadelphia, PA, USA
**University of California San Diego, La Jolla, CA, USA**	
Rany M Salem	Herbert Wertheim School of Public Health and Human Longevity Science, University of California San Diego, La Jolla, CA, USA
**University of Colorado School of Medicine, Aurora, CO, USA**	
Joanne B Cole	Department of Biomedical Informatics, University of Colorado School of Medicine, Aurora, CO, USA<br>Programs in Metabolism and Medical & Population Genetics, Broad Institute, Cambridge, MA USA.<br>Diabetes Unit and Center for Genomic Medicine, Massachusetts General Hospital, Boston, MA USA.

Supplementary Table S1: Number of cases and controls per phenotype definition

	Kidney failure vs normal AER			Kidney failure vs others			Severe DKD			Kidney failure vs. severe albuminuria			Severe albuminuria			Moderate albuminuria			Any DKD			CKD			CKD extremes			CKD+DKD		
**STUDY**	**N**	**Case**	**Ctrl**	**N**	**Case**	**Ctrl**	**N**	**Case**	**Ctrl**	**N**	**Case**	**Ctrl**	**N**	**Case**	**Ctrl**	**N**	**Case**	**Ctrl**	**N**	**Case**	**Ctrl**	**N**	**Case**	**Ctrl**	**N**	**Case**	**Ctrl**	**N**	**Case**	**Ctrl**
**Discovery**																														
FinnDiane	3669	974	2695	5679	974	4705	4232	1537	2695	1537	974	563	3258	563	2695	3524	829	2695	5061	2366	2695	3430	1068	2362	3337	975	2362	3347	890	2457
THLBB	794	70	724	2154	70	2084	842	118	724	118	70	48	772	48	724	826	102	724	944	220	724	281	73	208	278	70	208	355	23	332
LitDiane																60	21	39	79	40	39	71	21	50						
LatDiane							105	25	80							113	33	80	138	58	80									
RomDiane	117	28	89	235	28	207	187	98	89	98	28	70	159	70	89	137	48	89	235	146	89	220	53	167	195	28	167	126	39	87
UK-ROI	930	200	730	1396	200	1196	1434	704	730	666	200	466	1196	466	730				1434	704	730	1100	587	513	713	200	513	699	266	433
GWU GoKinD	572	261	311	601	261	340	601	290	311	290	261	29	340	29	311				601	290	311	598	273	325	586	261	325	578	269	309
France*	778	151	627	1071	151	920	959	332	627	332	151	181	806	181	625	749	124	625	1075	448	627	1021	281	740	899	159	740	803	225	578
Italy	303	142	161	343	142	201	341	180	161	180	142	38	199	38	161				343	180	163	329	157	172	323	148	175	309	155	154
Steno							902	488	414				883	469	414				916	489	427	890	200	690	718	28	690	504	106	398
Sweden	366	20	346	517	20	497	397	51	346	52	20	32	381	35	346	431	85	346	397	51	346	329	42	287	307	20	287	273	21	252
**Total Discovery**	7529	1846	5683	11996	1846	10150	10000	3823	6177	3273	1846	1427	7994	1899	6095	5840	1242	4598	11223	4992	6231	8269	2755	5514	7356	1889	5467	6994	1994	5000
**Replication**																														
Scotland				4689	57	4632	4157	195	3962										4689	727	3962	5116	404	4712	4792	80	4712	4540	90	4450
JOSLIN				2271	244	2027	1801	719	1082										2271	1189	1082	2107	533	1574	1957	262	1695	1415	402	1013
DCCT-EDIC				1305	23	1282	1100	84	1016										1305	289	1016	1298	80	1218	1241	23	1218	1046	47	999
CACTI				509	6	503	457	35	422										509	87	422	522	45	477	483	6	477	431	24	407
WESDR				556	104	452	510	217	293										556	263	293	605	207	398	502	104	398	400	140	260
EDC				418	59	359	327	134	193										418	225	193	418	130	288	347	59	288	269	89	180
**Total Replication**				**9748**	**493**	**9255**	**8352**	**1384**	**6968**										**9748**	**2780**	**6968**	**10066**	**1399**	**8667**	**9322**	**534**	**8788**	**8101**	**792**	**7309**

Case and control definitions for each DKD phenotype are specified in the **Supplementary Table S2**. AER: albumin excretion rate. *France: GENESIS and GENEDIAB study participants.

Supplementary Table S2: DKD phenotype case and control definitions

Phenotype name	Cases	Controls
**Kidney failure vs normal AER**	Kidney failure	Normal AER (AER < 20 µg/min or equivalent) and T1D duration ≥ 15 years
**Kidney failure vs others**	Kidney failure	No kidney failure
**Severe DKD**	Severe albuminuria (AER > 200 µg/min or equivalent) or kidney failure	Normal AER (AER < 20 µg/min or equivalent) and T1D duration ≥ 15 years
**Kidney failure vs. severe albuminuria**	Kidney failure	Severe albuminuria (AER > 200 µg/min or equivalent)
**Severe albuminuria**	Severe albuminuria (AER > 200 µg/min or equivalent)	Normal AER (AER < 20 µg/min or equivalent) and T1D duration ≥ 15 years
**Moderate albuminuria**	Moderate albuminuria (AER 20 - 200 µg/min or equivalent)	Normal AER (AER < 20 µg/min or equivalent) and T1D duration ≥ 15 years
**Any DKD**	Moderate or severe albuminuria (AER > 20 µg/min or equivalent) or kidney failure	Normal AER (AER < 20 µg/min or equivalent) and T1D duration ≥ 15 years
**CKD**	eGFR < 60 ml/min/1.73m^2	eGFR ≥ 60 ml/min/1.73m^2
**CKD extremes**	eGFR < 15 ml/min/1.73m^2	eGFR ≥ 90 ml/min/1.73m^2
**CKD+DKD**	eGFR < 45 ml/min/1.73m^2 AND (moderate or severe albuminuria (AER > 20 µg/min or equivalent) or kidney failure)	eGFR ≥ 60 ml/min/1.73m^2 AND normal AER (AER < 20 µg/min or equivalent) and T1D duration ≥ 15 years

Supplementary table S3: Genotype counts for *EXD3* rs200080727 across the 3 discovery cohorts.

Cohort	Stratum	CC	CT	TT	P HWE	Beta (se)	P
Italy	Cases	153	≤3	0	1		
	Controls	154	≤3	0	1		
	Total	307	≤3	0	1	13.1 (2.51)	7.1×10^{-6}
Steno	Cases	103	≤3	0	1		
	Controls	398	≤3	0	1		
	Total	501	≤3	0	1	13.6 (1.67)	3.9×10^{-5}
Sweden	Cases	20	≤3	0	1		
	Controls	250	≤3	0	1		
	Total	270	≤3	0	1	-0.34 (11.76)	0.978
		**CC**	**CT**	**TT**	**Crude OR**	**Beta (se)**	**P**
Meta-analysis total	Cases	276	6	0			
	Controls	802	2	0			
	Total	1078	8	0	8.7	8.1 (1.38)	4.5×10^{-9}

Exact genotype counts ≤3 per study are not reported to protect the privacy of those carrying the rare variant. Beta (se) and P: effect size estimate and *p*-value from score test for individual cohorts, or from the inverse variance weighted meta-analysis. Crude OR: unadjusted crude odds ratio per T allele (i.e. for CT vs CC) based on total allele counts.

Supplementary Table S4: *EXD3* p.Asp555Asn (rs200080727) association across the 10 phenotype definitions in the discovery cohort.

Phenotype	N	AF	Direction	Effect size	P
CKD+DKD	1086	0.0037	??????++-	8.102	4.46×10^{-9}
CKD	1548	0.0026	???????++-	6.155	5.41×10^{-7}
CKD extremes	630	0.0040	??????+?-	10.846	1.08×10^{-5}
Kidney failure vs others	860	0.0029	??????++	6.132	1.32×10^{-4}
Any DKD	1656	0.0024	????????+++	3.032	6.36×10^{-4}
Moderate albuminuria	431	0.0023	??????-	-1.164	0.63
Severe DKD	1640	0.0024	???????+++	3.003	7.00×10^{-4}
Severe albuminuria	1264	0.0020	???????+-	1.768	0.10
Kidney failure vs normal AER	669	0.0037	??????++	5.617	5.74×10^{-4}
Kidney failure vs severe albuminuria	180	0.0056	??????+?	2.280	0.19

AF: Allele frequency. Direction: direction of effect (for the alternative allele) in each contributing study: + increased risk, - decreased risk, ? not available/detected. The final 3 studies with +/- effects are Italy, Steno, and the Sweden cohorts.

Supplementary Table S5: Clinical characteristics of the *EXD3* rs200080727 T allele carriers

Variable	Mean Non-carriers	N Non-carriers	Mean Carriers	N Carriers	P-value
DBP [mmHg]	77.9	2655	89.8	9	**0.004**
eGFR [ml/min/1.73m^2]	81.0	2490	36.3	8	**0.009**
SBP [mmHg]	137.1	2655	150.6	9	**0.019**
HbA1c [%]	8.6	2652	9.6	9	0.170
HbA1c [mmol/mol]	70.5	2652	81.4	9	0.170
BMI [kg/m^2]	24.7	2489	25.1	8	0.779

Clinical characteristics of the *EXD3* rs200080727 T allele carriers, pooled over the Steno, Italian, and Swedish cohort participants with type 1 diabetes. Values are given as mean. Group means are compared with t-test.

Supplementary Table S6: Lead gene non-synonymous variants with P-value <0.05 in discovery or replication stage

						Discovery				Replication*				Variant Annotation		
GENE	Phenotype	Variant	Location	REF	ALT	MAF	MAC	BETA	P-value	MAF	MAC	Beta	P-value		SIFT	PolyPhen
COL4A3	Severe DKD	rs55703767	2:228121101	G	T	0.201	4017	-0.226	5.00×10^{-8}	0.213	3563	-0.289	1.25×10^{-6}	p.D326Y	tolerated(0.06)	benign(0.157)
EOMES	Any DKD	rs200789175	3:27763770	G	C	0.006	116	0.981	2.67×10^{-6}	0.012	215	0.084	0.644	p.Q6E	tolerated_low_confidence (0.44)	benign(0.05)
EXD3	CKD+DKD	rs200080727	9:140243729	C	T	0.004	8	8.100	4.46×10^{-9}					p.D555N	**deleterious(0)**	**probably_damaging(1)**
EXD3	CKD+DKD	rs28545754	9:140243844	T	C	0.361	1013	-0.210	0.022	0.367	4293	0.076	0.478	p.Y545C	**deleterious(0.02)**	benign(0.015)
EXD3	CKD+DKD	rs35097575	9:140243880	G	A	0.062	825	0.177	0.045	0.056	914	0.225	0.093	p.T533M	**deleterious(0.03)**	benign(0.198)
EXD3	CKD+DKD	rs201678237	9:140250729	C	T	0.0003	≤3	3.626	0.036					p.V250I	tolerated(0.16)	benign(0.006)
EXD3	CKD+DKD	rs116575725	9:140243973	G	A					0.004	12	3.054	0.015	p.A502V	tolerated(0.35)	benign(0.007)
EXD3	CKD+DKD	rs115998217	9:140247103	C	T					0.006	41	1.536	0.006	p.V336M	tolerated(0.1)	benign(0.254)
IGSF3	Kidney failure vs others	rs749817295	1:117208941	C	T	0.001	6	10.750	2.49×10^{-10}					p.C3Y	**deleterious(0)**	**probably_damaging(0.974)**
KIAA1109	Any DKD	rs74643640	4:123113428	A	G	0.021	463	-0.396	1.79×10^{-4}	0.035	678	-0.139	0.153	p.S316G	tolerated(0.17)	benign(0)
KIAA1109	Any DKD	rs2306369	4:123268859	A	G	0.022	488	-0.381	2.34×10^{-4}	0.036	706	-0.103	0.284	p.T4352A	tolerated(0.68)	benign(0)
LAIR1	Kidney failure vs others	rs140068764	19:54872657	G	C	0.002	10	6.113	1.74×10^{-6}					p.A70G	tolerated(0.4)	benign(0.03)
MUC5B	CKD	rs199977970	11:1258377	G	A	0.004	25	1.017	0.039					p.A1094T	**deleterious(0.01)**	**probably_damaging(0.949)**
MUC5B	CKD	rs78692183	11:1263757	A	G	0.048	802	0.191	0.031	0.033	668	-0.215	0.095	p.S1883G	tolerated(0.22)	benign(0.007)
MUC5B	CKD	rs183657141	11:1263932	C	G	0.030	270	0.347	0.019	0.033	668	-0.215	0.095	p.T1941S	tolerated(0.4)	benign(0.019)
MUC5B	CKD	rs2943506	11:1264160	C	T	0.030	270	0.351	0.017	0.033	672	-0.214	0.095	p.A2017V	tolerated(0.3)	benign(0.001)
MUC5B	CKD	rs543947285	11:1266275	C	T	0.029	266	0.361	0.017	0.033	654	-0.235	0.075	p.T2722I	tolerated(0.11)	benign(0.007)
MUC5B	CKD	rs116870693	11:1267120	G	A	0.030	271	0.333	0.024	0.034	674	-0.218	0.089	p.A3004T	tolerated(0.34)	benign(0)
MUC5B	CKD	rs201236828	11:1267250	C	A	0.030	270	0.347	0.019	0.033	668	-0.215	0.094	p.T3047N	tolerated(0.07)	possibly_damaging(0.651)
MUC5B	CKD	rs188370271	11:1267253	C	T	0.030	270	0.347	0.019	0.033	668	-0.215	0.094	p.A3048V	tolerated(0.52)	benign(0.089)
MUC5B	CKD	rs184565965	11:1267289	A	C	0.049	805	0.186	0.035	0.033	668	-0.215	0.094	p.K3060T	tolerated(0.89)	benign(0)
MUC5B	CKD	rs189329205	11:1267291	T	C	0.049	805	0.186	0.035	0.033	668	-0.215	0.094	p.S3061P	tolerated(0.21)	benign(0)
MUC5B	CKD	rs60867894	11:1267372	A	G	0.031	304	0.347	0.013	0.033	670	-0.220	0.084	p.M3088V	tolerated(0.43)	benign(0)
MUC5B	CKD	rs200012359	11:1267640	C	G	0.034	105	0.322	0.223	0.032	180	-0.634	0.006	p.T3177S	tolerated(0.19)	benign(0.246)
MUC5B	CKD	rs117913875	11:1267688	T	C	0.031	304	0.345	0.013	0.033	668	-0.215	0.094	p.L3193P	tolerated(0.3)	unknown(0)
MUC5B	CKD	rs184424771	11:1269770	C	T	0.029	268	0.358	0.016	0.033	668	-0.215	0.095	p.P3887L	tolerated(0.58)	benign(0.007)
MUC5B	CKD	rs201532622	11:1270361	C	T	0.030	270	0.357	0.017	0.033	660	-0.219	0.092	p.T4084M	**deleterious(0.04)**	possibly_damaging(0.454)
MUC5B	CKD	rs200302235	11:1271060	A	C	0.030	270	0.347	0.019	0.033	668	-0.215	0.094	p.K4317T	tolerated(0.58)	benign(0)
MUC5B	CKD	rs201332455	11:1271062	T	C	0.030	270	0.347	0.019	0.033	668	-0.215	0.094	p.S4318P	tolerated(0.17)	benign(0)
MUC5B	CKD	rs201326462	11:1271077	G	T	0.030	270	0.347	0.019	0.033	668	-0.215	0.094	p.V4323F	tolerated(0.77)	benign(0)
MUC5B	CKD	rs200027230	11:1271485	G	A	0.005	≤3	3.561	0.012					p.V4459M	tolerated(0.22)	benign(0)

						Discovery				Replication*				Variant Annotation		
GENE	Phenotype	Variant	Location	REF	ALT	MAF	MAC	BETA	P-value	MAF	MAC	Beta	P-value		SIFT	PolyPhen
MUC5B	CKD	rs4046524	11:1271891	C	T	0.031	303	0.353	0.012	0.033	668	-0.216	0.094	p.P4597L	-	benign(0)
MUC5B	CKD	rs191989562	11:1272694	G	A	0.030	270	0.347	0.019	0.033	668	-0.215	0.094	p.G4862S	tolerated(0.78)	benign(0.107)
MUC5B	CKD	rs56232219	11:1275988	T	C	0.048	802	0.191	0.031	0.033	668	-0.215	0.095	p.L5181P	tolerated(0.05)	benign(0.003)
MUC5B	CKD	rs55657020	11:1278796	G	A	0.048	802	0.191	0.031	0.033	668	-0.216	0.093	p.V5436M	tolerated (0.08)	benign (0.177)
MUC5B	CKD	rs56220864	11:1278912	G	C	0.048	802	0.192	0.030	0.033	668	-0.216	0.093	p.E19D	tolerated (1)	benign (0)
MUC5B	CKD extremes	rs10835639	11:1262312	G	A	0.006	43	-0.304	0.446	0.002	4	8.134	0.007	p.R1401H	tolerated (0.39)	benign (0.007)
MUC5B	CKD extremes	rs183657141	11:1263932	C	G	0.028	209	0.472	0.025	0.032	600	-0.073	0.718	p.T1941S	tolerated (0.4)	benign (0.019)
MUC5B	CKD extremes	rs2943506	11:1264160	C	T	0.028	209	0.485	0.021	0.032	604	-0.075	0.713	p.A2017V	tolerated (0.3)	benign (0.001)
MUC5B	CKD extremes	rs543947285	11:1266275	C	T	0.028	206	0.492	0.023	0.032	587	-0.101	0.626	p.T2722I	tolerated (0.11)	benign (0.007)
MUC5B	CKD extremes	rs116870693	11:1267120	G	A	0.028	209	0.461	0.029	0.033	606	-0.073	0.718	p.A3004T	tolerated (0.34)	benign (0)
MUC5B	CKD extremes	rs201236828	11:1267250	C	A	0.028	209	0.472	0.025	0.032	600	-0.074	0.718	p.T3047N	tolerated (0.07)	possibly_damaging (0.651)
MUC5B	CKD extremes	rs188370271	11:1267253	C	T	0.028	209	0.472	0.025	0.032	600	-0.074	0.718	p.A3048V	tolerated (0.52)	benign (0.089)
MUC5B	CKD extremes	rs60867894	11:1267372	A	G	0.030	243	0.458	0.016	0.032	600	-0.085	0.671	p.M3088V	tolerated (0.43)	benign (0)
MUC5B	CKD extremes	rs117913875	11:1267688	T	C	0.030	243	0.456	0.016	0.032	600	-0.073	0.718	p.L3193P	tolerated (0.3)	unknown (0)
MUC5B	CKD extremes	rs184424771	11:1269770	C	T	0.028	207	0.484	0.023	0.032	602	-0.074	0.718	p.P3887L	tolerated (0.58)	benign (0.007)
MUC5B	CKD extremes	rs201532622	11:1270361	C	T	0.028	209	0.485	0.023	0.032	595	-0.072	0.727	p.T4084M	**deleterious (0.04)**	possibly_damaging (0.454)
MUC5B	CKD extremes	rs200302235	11:1271060	A	C	0.028	209	0.472	0.025	0.032	600	-0.074	0.718	p.K4317T	tolerated (0.58)	benign (0)
MUC5B	CKD extremes	rs201332455	11:1271062	T	C	0.028	209	0.472	0.025	0.032	600	-0.074	0.718	p.S4318P	tolerated (0.17)	benign (0)
MUC5B	CKD extremes	rs201326462	11:1271077	G	T	0.028	209	0.473	0.025	0.032	600	-0.074	0.716	p.V4323F	tolerated (0.77)	benign (0)
MUC5B	CKD extremes	rs200027230	11:1271485	G	A	0.005	≤3	3.665	0.010					p.V4459M	tolerated (0.22)	benign (0)
MUC5B	CKD extremes	rs4046524	11:1271891	C	T	0.030	242	0.467	0.014	0.032	600	-0.075	0.711	p.P4597L	-	benign (0)
MUC5B	CKD extremes	rs191989562	11:1272694	G	A	0.028	209	0.472	0.025	0.032	600	-0.074	0.718	p.G4862S	tolerated (0.78)	benign (0.107)
MUC5B	CKD extremes	rs200912848	11:1270892	C	A					0.002	5	5.141	0.040	p.A4261E	tolerated (1)	benign (0.013)
MUC5B	CKD extremes	rs201463531	11:1271180	C	T					0.0004	4	20.724	$9.42{\times}10^{-6}$	p.P4357L	**deleterious (0.01)**	benign (0.086)
PLCB2	Kidney failure vs others	rs779144751	15:40581092	C	T	0.002	9	4.250	0.002					p.E17K	**deleterious_low_confidence (0.05)**	benign (0.018)
PLCB2	Kidney failure vs others	rs199647108	15:40594758	A	C	0.002	31	1.779	$1.60{\times}10^{-4}$					p.L95R	tolerated (0.1)	possibly_damaging (0.883)
ZAN	CKD extremes	rs200690150	7:100348890	G	T	0.001	8	1.712	0.047					splice donor	-	-
ZAN	CKD extremes	rs141127397	7:100377138	A	AGGGC	0.008	110	0.648	0.007	0.005	103	-0.650	0.173	p.A2129AGX	-	-
ZAN	CKD extremes	rs149104440	7:100389677	C	T	0.019	199	0.667	$1.35{\times}10^{-4}$	0.025	323	-0.359	0.336	p.R2540*	-	-

*Replication in GWAS on DKD in T1D from Scotland, Joslin Diabetes Center, DCCT study, EDC, CACTI, and WESDR studies. Variant associations with p<0.05 are highlighted with green color.

Supplementary Table S7: *EXD3* p.Asp555Asn (rs200080727) association and genotype validation with sequencing data in the FinnDiane cohort subset.

A: Association with CDK+DKD in the FinnDiane cohort genotyping batch 1 (B1) genotyped with Illumina HumanCoreExome bead array 12-1.0. B: genotype validation with whole-exome (WES) and whole-genome sequencing (WGS) data

A:

	FinnDiane B1				
	**CC**	**CT**	**TT**	**Beta**	**P**
**Cases**	1007	≤3	0		
**Controls**	1767	8	0		
**Total**	2774	≤11	0	-0.27	0.095

B:

		FinnDiane GWAS, B1		
		**CC**	**CT**	**TT**
**FinnDiane WES and WGS**	**CC**	1990	0	0
	**CT**	0	6	0
	**TT**	0	0	0

Whole exome and whole genome sequencing (WES, WGS) data were available for 2,354 partially overlapping FinnDiane study participants, similar to our previously published WES/WGS data for N=1064 participants.[S7] CKD+DKD phenotype: cases with albuminuria (AER ≥ 20 mg/min or equivalent) and eGFR ≤45 ml/min/1.73m^2; controls with normal albumin excretion (AER < 20 mg/min or equivalent) and eGFR ≥ 60 ml/min/1.73m^2 and T1D duration ≥ 15 years.

Supplementary Table S8: *EXD3* p.Asp555Asn (rs200080727) replication in the UK Biobank individuals with type 2 diabetes.

Phenotype	Variant	Estimate	Std. Error	Pr(>|t|)
Albuminuria*	rs200080727_T	41.78	16.91	**0.014**
eGFR*	rs200080727_T	-0.58	1.29	0.65
CKD+DKD	rs200080727_T	0.15	0.72	0.84

Analysis included N=19,191 individuals with type 2 diabetes, with *EXD3* p.Asp555Asn minor allele count (MAC) of 158 (MAF 0.4%). *Albuminuria and eGFR were analysed as continuous traits. CKD+DKD phenotype: cases with albuminuria (AER ≥ 20 mg/min or equivalent) and eGFR ≤45 ml/min/1.73m^2; controls with normal albumin excretion (AER < 20 mg/min or equivalent) and eGFR ≥ 60 ml/min/1.73m^2 and T1D duration ≥ 15 years.

Supplementary Table S9: *EXD3* and *MUC5B* gene expression in mRNA sequencing studies.

Dataset	Analysis	p-Value	Fold Change	Reporter	N Controls	N cases	Kidney compartment
EXD3							
Nakagawa CKD Kidney[S8]	Chronic Kidney Disease vs. Normal Kidney (Discovery Set)	**4.95×10^{-14}**	**-2.59**	A_24_P49183	5	48	whole kidney
Nakagawa CKD Kidney[S8]	Chronic Kidney Disease vs. Normal Kidney (Validation Set)	**7.26×10^{-5}**	**-4.41**	A_24_P49183	3	5	whole kidney
Woroniecka Diabetes TubInt[S9]	Diabetic Nephropathy vs. Healthy Living Donor	**0.007**	-1.36	220838_at	12	10	TubInt
Re-analysis of Levin et al 2020[S10]	DN vs Healthy Control	0.07	1.07		20	19	Tubular
Re-analysis of Fan et al 2019[S11]	Advanced DN vs Controls	0.17	1.19		9	22	whole kidney
Re-analysis of Fan et al 2019[S11]	Early DN vs Controls	0.44	1.56		9	6	whole kidney
Schmid Diabetes TubInt[S12]	Diabetic Nephropathy vs. Minimal Change Disease and Control	0.48	1.05	220838_at	11	11	TubInt
Schmid Diabetes TubInt[S12]	Diabetic Nephropathy vs. Control	0.48	1.06	220838_at	7	11	TubInt
Re-analysis of Fan et al 2019[S11]	Advanced DN vs Early DN	0.76	2.14		6	22	whole kidney
ERCB microarray data	Diabetic nephropathy (DN) vs living donors	0.36	1.01		46	17	TubularInt
ERCB microarray data	Diabetic nephropathy (DN) vs living donors	0.78	0.99		48	12	Glomerular
Schmid Diabetes TubInt[S12]	Minimal Change Disease vs. Control	0.81	1.02	220838_at	7	4	TubInt
Woroniecka Diabetes Glom[S9]	Diabetic Nephropathy vs. Healthy Living Donor	0.86	1.02	220838_at	13	9	Glom
Re-analysis of Levin et al 2020[S10]	DN vs Healthy Control	0.97	2.65		20	19	Glomerular
MUC5B							
Re-analysis of Fan et al 2019 [S11]	Advanced DN vs Control	**2.19×10^{-6}**	-1.19		9	21	whole kidney
Nakagawa CKD Kidney [S8]	Chronic Kidney Disease vs. Normal Kidney (Discovery Set)	**3.34×10^{-5}**	**3.18**	A_23_P305863	5	48	whole kidney
Re-analysis of Fan et al 2019 [S11]	Advanced DN vs Early DN	**0.002**	-1.13		6	21	whole kidney
Woroniecka Diabetes TubInt[S9]	Diabetic Nephropathy vs. Healthy Living Donor	**0.011**	-1.18		12	10	TubInt
Nakagawa CKD Kidney [S8]	Chronic Kidney Disease vs. Normal Kidney (Validation Set)	**0.014**	**6.18**	213432_at	3	5	whole kidney
Ju CKD TubInt[S13]	Diabetic Nephropathy vs. Healthy Living Donor	0.07	-1.07	A_23_P305863	31	17	TubInt
ERCB microarray data	Diabetic nephropathy (DN) vs living donors	0.08	-1.08	727897	48	12	Glomerular
Ju CKD Glom[S13]	Diabetic Nephropathy vs. Healthy Living Donor	0.09	-1.12	727897	21	12	Glomerular
Woroniecka Diabetes Glom[S9]	Diabetic Nephropathy vs. Healthy Living Donor	0.09	1.17	222268_x_at	13	9	Glomerular
Re-analysis of Fan et al 2019 [S11]	Early DN vs Control	0.16	-1.05	213432_at	9	6	whole kidney
ERCB microarray data	Diabetic nephropathy (DN) vs living donors	0.23	-1.05	213432_at	46	17	TubularInt
Schmid Diabetes TubInt [S12]	Diabetic Nephropathy vs. Minimal Change Disease and Control	0.29	-1.05	213432_at	11	11	TubInt
Schmid Diabetes TubInt [S12]	Diabetic Nephropathy vs. Control	0.47	-1.04		7	11	TubInt
Schmid Diabetes TubInt [S12]	Minimal Change Disease vs. Control	0.51	1.03		7	4	TubInt

Differential gene expression analysis results for Nakagawa CKD Kidney[S8], Woroniecka Diabetes[S9], Schmid Diabetes[S12], and Ju CKD TubIng/Glom[S13] data sets were queried from the Nephroseq portal (v5.nephroseq.org). mRNA sequence data from ERCB microarray data (Supplementary Table S11), Levin et al 2020, [S10] and Fan et al 2019[S11] datasets were reanalysed as described in the supplementary methods.[S14] Significant p-values ($p<0.05$) and Fold changes ($|FC|>2$) are highlighted with bold.

Supplementary Table S10: *EXD3* gene expression in single nucleus (snRNA) and single cell (scRNA) sequencing data of >200,000 cells from human normal and disease kidneys.

Cell population	Average Expression (All Samples)	Fraction Expressing (All Samples)	CellType marker p-values	Average expression			Fraction Expressing			CKD vs. Control	
				Control Samples	AKI Samples	CKD Samples	Control Samples	AKI Samples	CKD Samples	p-value	Fold Change
**snRNA**											
iPT	0.36	0.26	**1.20E-61**	0.36	0.41	0.33	0.27	0.31	0.24	**1.30E-02**	-1.09
ATL	0.38	0.32	**2.20E-47**	0.35	0.43	0.43	0.32	0.39	0.33	**1.10E-21**	1.23
C_TAL	0.31	0.25	**5.80E-21**	0.3	0.34	0.32	0.25	0.3	0.25	NS	
PC	0.34	0.27	**4.70E-18**	0.32	0.36	0.34	0.27	0.27	0.25	**1.40E-04**	1.06
Endo_GC	0.4	0.25	**9.80E-18**	0.37	0.36	0.43	0.24	0.22	0.25	NS	
Podo	0.32	0.26	**4.60E-08**	0.33	0.34	0.31	0.26	0.32	0.25	NS	
Endo_Peritubular	0.32	0.19	**3.20E-05**	0.32	0.33	0.34	0.18	0.18	0.2	NS	
PT_S1	0.34	0.23	**1.70E-02**	0.35	0.35	0.32	0.24	0.25	0.19	**3.50E-02**	-1.09
PT_S2	0.34	0.26	NS	0.3	0.44	0.28	0.17	0.41	0.22	NS	
Macula_Densa	0.32	0.25	NS	0.28	0.4	0.32	0.22	0.32	0.24	**3.10E-02**	1.14
PEC	0.32	0.2	NS	0.31	0.34	0.33	0.19	0.22	0.21	NS	
CNT	0.31	0.26	NS	0.31	0.32	0.31	0.26	0.27	0.24	NS	
DCT1	0.31	0.25	NS	0.29	0.32	0.32	0.23	0.26	0.24	NS	
DTL	0.31	0.23	NS	0.28	0.33	0.32	0.2	0.29	0.23	**1.50E-02**	1.14
M_TAL	0.31	0.24	NS	0.32	0.32	0.3	0.25	0.24	0.22	**3.00E-08**	-1.07
DCT2	0.29	0.22	NS	0.29	0.37	0.28	0.22	0.3	0.2	NS	
IC_A	0.28	0.23	NS	0.28	0.35	0.31	0.23	0.26	0.24	NS	
Neural_Cells	0.28	0.16	NS	0.27	0.23	0.3	0.17	0.14	0.15	NS	
cDC	0.27	0.16	NS	0.29	0.29	0.21	0.18	0.16	0.12	NS	
B_memory	0.26	0.15	NS	0.14	0.38	0.27	0.09	0.23	0.16	NS	
Enod_Lym	0.25	0.17	NS	0.28	0.21	0.22	0.19	0.1	0.15	NS	
IC_B	0.25	0.17	NS	0.22	0.29	0.25	0.15	0.21	0.18	NS	
CD16_Mono	0.24	0.14	NS	0.22	NS	0.2	0.12	NS	0.12	NS	
PT_S3	0.24	0.16	NS	0.24	0.22	0.26	0.16	0.16	0.16	NS	
Fib	0.22	0.14	NS	0.16	0.18	0.28	0.11	0.13	0.16	NS	
GS_Stromal	0.22	0.11	NS	0.23	0.27	0.2	0.12	0.16	0.1	NS	
VSMC/Pericyte	0.22	0.12	NS	0.21	0.21	0.23	0.12	0.12	0.12	NS	
Mac	0.21	0.12	NS	0.2	0.26	0.21	0.12	0.14	0.11	NS	
Mes	0.2	0.12	NS	0.19	0.26	0.21	0.12	0.17	0.12	NS	
Neutrophil	0.2	0.13	NS	0.14	0.21	0.25	0.09	0.12	0.16	NS	
MyoFib	0.17	0.11	NS	0.18	0.19	0.16	0.11	0.11	0.09	NS	
Baso/Mast	0.15	0.08	NS	0.22	NS	NS	0.11	NS	NS	NS	
CD8T	0.14	0.08	NS	0.11	0.12	0.19	0.05	0.08	0.1	NS	
NK	0.14	0.08	NS	0.05	0.15	0.17	0.04	0.07	0.09	NS	
B_Naive	0.13	0.07	NS	0.11	0.18	0.14	0.05	0.1	0.07	NS	
CD4T	0.13	0.08	NS	0.11	0.14	0.17	0.06	0.1	0.1	NS	
Plasma_Cells	0.13	0.08	NS	0.19	0.11	0.1	0.12	0.08	0.07	NS	
CD14_Mono	0.1	0.06	NS	0.03	0.11	0.15	0.03	0.08	0.07	NS	
pDC	0.1	0.05	NS	0.13	NS	0.06	0.06	NS	0.05	NS	
B_Cells	NS	NS	NS	NS	NS	NS	NS	NS	NS	NS	
DCT	NS	NS	NS	NS	NS	NS	NS	NS	NS	NS	
DLOH	NS	NS	NS	NS	NS	NS	NS	NS	NS	NS	
EC	NS	NS	NS	NS	NS	NS	NS	NS	NS	NS	
Endo_Lymphatic	NS	NS	NS	NS	NS	NS	NS	NS	NS	NS	
FIB	NS	NS	NS	NS	NS	NS	NS	NS	NS	NS	
Fibroblast	NS	NS	NS	NS	NS	NS	NS	NS	NS	NS	
IC	NS	NS	NS	NS	NS	NS	NS	NS	NS	NS	
IMM	NS	NS	NS	NS	NS	NS	NS	NS	NS	NS	
NA	NS	NS	NS	NS	NS	NS	NS	NS	NS	NS	
NEU	NS	NS	NS	NS	NS	NS	NS	NS	NS	NS	
PapE	NS	NS	NS	NS	NS	NS	NS	NS	NS	NS	
POD	NS	NS	NS	NS	NS	NS	NS	NS	NS	NS	

PT	NS	NS	NS	NS	NS	NS	NS	NS	NS	NS
TAL	NS	NS	NS	NS	NS	NS	NS	NS	NS	NS
VSM/P	NS	NS	NS	NS	NS	NS	NS	NS	NS	NS
**scRNA**										
iPT	0.08	0.08	3.30E-22	0.06	0.11	0.07	0.07	0.11	0.07	NS
IC_A	0.08	0.1	5.30E-18	0.08	0.1	0.07	0.11	0.11	0.08	NS
CNT	0.07	0.07	1.10E-14	0.06	0.09	0.07	0.06	0.09	0.06	NS
PC	0.09	0.1	5.90E-07	0.08	0.12	0.07	0.11	0.12	0.07	NS
VSMC/Pericyte	0.1	0.07	3.30E-03	0.1	0.12	0.09	0.08	0.08	0.06	NS
M_TAL	0.06	0.06	1.40E-02	0.04	0.06	0.07	0.05	0.06	0.06	NS
ATL	0.08	0.08	1.70E-02	NS	0.09	0.07	NS	0.08	0.06	NS
DCT2	0.07	0.07	2.10E-02	0.04	0.12	0.07	0.05	0.11	0.06	NS
Macula_Densa	0.06	0.07	3.10E-02	0.05	0.07	0.06	0.06	0.07	0.06	NS
C_TAL	0.05	0.05	4.90E-02	0.04	0.06	0.05	0.05	0.06	0.04	NS
Podo	0.11	0.09	NS	0.15	0.12	0.03	0.12	0.08	0.03	NS
PEC	0.1	0.11	NS	0.14	0.07	0.06	0.16	0.08	0.06	NS
Endo_GC	0.09	0.06	NS	0.08	0.11	0.08	0.06	0.07	0.05	NS
Endo_Peritubular	0.07	0.04	NS	0.05	0.09	0.07	0.04	0.06	0.04	NS
DTL	0.06	0.06	NS	0	0.07	0.07	0	0.06	0.06	NS
Enod_Lym	0.06	0.04	NS	NS	0	0.09	NS	0	0.05	NS
Mes	0.06	0.04	NS	0.06	0.03	0.07	0.03	0.02	0.04	NS
DCT1	0.05	0.05	NS	0.05	0.06	0.04	0.06	0.06	0.04	NS
Fib	0.05	0.05	NS	0	0.04	0.06	0	0.03	0.06	NS
IC_B	0.05	0.06	NS	0.05	0.05	0.05	0.07	0.05	0.06	NS
PT_S3	0.05	0.05	NS	0.04	0.07	0.06	0.03	0.07	0.05	NS
Baso/Mast	0.04	0.02	NS	0.05	NS	0.03	0.02	NS	0.02	NS
Mac	0.04	0.02	NS	0.03	0.04	0.04	0.02	0.03	0.03	NS
MyoFib	0.04	0.03	NS	0.02	0.08	0.02	0.02	0.04	0.02	NS
NK	0.04	0.02	NS	0.03	0.07	0.04	0.02	0.04	0.02	NS
CD4T	0.03	0.02	NS	0.03	0.02	0.03	0.02	0.01	0.02	NS
CD8T	0.03	0.02	NS	0.03	0.03	0.02	0.02	0.02	0.02	NS
cDC	0.03	0.02	NS	0.02	0.03	0.03	0.02	0.02	0.03	NS
pDC	0.03	0.02	NS	0	0.02	0.05	0	0.02	0.03	NS
PT_S1	0.03	0.02	NS	0.03	0.05	0.02	0.02	0.04	0.02	NS
B_memory	0.02	0.02	NS	0.02	0.02	0.03	0.01	0.01	0.02	NS
CD16_Mono	0.02	0.01	NS	0	0.06	0.01	0	0.04	0.01	NS
GS_Stromal	0.02	0.02	NS	0.01	NS	0.04	0	NS	0.03	NS
Neutrophil	0.02	0.01	NS	0	0.02	0.02	0	0.02	0.01	NS
Plasma_Cells	0.02	0.02	NS	0	0.02	0.02	0	0.01	0.02	NS
PT_S2	0.02	0.02	NS	0.01	0.05	0.03	0.01	0.04	0.02	NS
B_Naive	0.01	0.01	NS	0.02	0	0.01	0.02	0	0.01	NS
CD14_Mono	0.01	0.01	NS	0.01	0	0.01	0.01	0	0.01	NS
Neural_Cells	0	0	NS	0	NS	0	0	NS	0	NS
B_Cells	NS	NS	NS	NS	NS	NS	NS	NS	NS	NS
DCT	NS	NS	NS	NS	NS	NS	NS	NS	NS	NS
DLOH	NS	NS	NS	NS	NS	NS	NS	NS	NS	NS
EC	NS	NS	NS	NS	NS	NS	NS	NS	NS	NS
Endo_Lymphatic	NS	NS	NS	NS	NS	NS	NS	NS	NS	NS
FIB	NS	NS	NS	NS	NS	NS	NS	NS	NS	NS
Fibroblast	NS	NS	NS	NS	NS	NS	NS	NS	NS	NS
IC	NS	NS	NS	NS	NS	NS	NS	NS	NS	NS
IMM	NS	NS	NS	NS	NS	NS	NS	NS	NS	NS
NA	NS	NS	NS	NS	NS	NS	NS	NS	NS	NS
NEU	NS	NS	NS	NS	NS	NS	NS	NS	NS	NS
PapE	NS	NS	NS	NS	NS	NS	NS	NS	NS	NS
POD	NS	NS	NS	NS	NS	NS	NS	NS	NS	NS
PT	NS	NS	NS	NS	NS	NS	NS	NS	NS	NS
TAL	NS	NS	NS	NS	NS	NS	NS	NS	NS	NS
VSM/P	NS	NS	NS	NS	NS	NS	NS	NS	NS	NS

Data from susztaklab.com.[S15] Log and normalized average expression was calculated across all samples (Average Expression (All Samples)), across just control samples Average Expression (Control Samples), and across just diseased samples Average Expression (Disease Samples). P-values for differentially expressed in each cell type (Celltype marker P-values) and for differentially expressed in disease samples as compared to control samples were calculated using a Wilcoxon rank-sum test and were corrected for multiple testing using the Benjamini-Hochberg method (adjusted for testing 34,733 genes). iPT: injured proximal tubules. C_TAL and M_TAL, cortical and medullary thick ascending loop of Henle. PC, principal cells of collecting duct. Podo, podocytes. PT_S1, Proximal tubule subtype 1. Endo_GC, endothelial cells of glomerular capillary tuft. IC_A, type alpha intercalated cells.

Supplementary Table S11: Differential gene expression in the ERCB microarray data for diabetic nephropathy vs living donors.

Tissue	Gene	logFC	Average Expression	t	*P*-value	Adjusted P
Glomerular	*LAIR1*	0.613596	6.822	4.259	7.13×10^{-5}	**8.98×10^{-4}**
Glomerular	*IGSF3*	0.214005	7.582	2.560	0.0130	**0.0475**
Glomerular	*KIAA1109*	-0.09472	6.174	-2.276	0.0263	0.0806
Glomerular	*PLCB2*	-0.10922	6.540	-1.958	0.0548	0.1363
Glomerular	*MUC5B*	-0.11703	5.043	-1.802	0.0764	0.1731
Glomerular	*EXD3*	-0.01722	6.013	-0.279	0.7814	0.8572
TubularInt	*IGSF3*	0.24426001	7.103	3.908	2.24×10^{-4}	**0.0016**
TubularInt	*LAIR1*	0.22417823	5.844	2.943	0.0045	**0.0170**
TubularInt	*PLCB2*	-0.1212199	5.897	-2.112	0.0386	0.0895
TubularInt	*MUC5B*	-0.065788	4.728	-1.208	0.2313	0.3519
TubularInt	*EXD3*	-0.044141	4.928	-0.928	0.3568	0.4855
TubularInt	*KIAA1109*	0.01007641	5.612	0.287	0.7752	0.8433

LogFC: log fold change. Adjusted P: corrected for 6 tested genes × 2 tested tissues. TubularInt: tubular and interstitial tissue. Glomerular tissue N: 12 diabetic nephropathy samples vs 48 living donor samples. Tubular tissue N: 17 diabetic nephropathy samples, vs 46 living donor samples.

Supplementary Table S12: Lead gene associations across the ten DKD definitions. Results are shown for the same variant category (non-synonymous/ protein-truncating variants), test (SKAT, burden, VT), and MAF cut-off (5%, 1%, or 0.5%) as the original gene aggregate test result with smallest p-value.

Gene	Var types	Test	MAF cut-off	Phenotype	N var	*p*-value	N total
EOMES	Non-synonymous	SKAT	0.05	Any DKD	2	**1.1E-06**	11223
				Severe DKD	2	**2.8E-05**	10000
				Severe albuminuria	2	**5.6E-04**	7994
				CKD+DKD	2	**0.002**	6994
				Kidney failure vs normal AER	2	**0.003**	7529
				Moderate albuminuria	2	**0.004**	5840
				CKD	2	**0.033**	8269
				Kidney failure vs others	3	0.091	11996
				CKD extremes	2	0.095	7356
				Kidney failure vs severe albuminuria	2	0.756	3273
IGSF3	Non-synonymous	SKAT	0.01	Kidney failure vs others	2	**1.5E-10**	11996
				Kidney failure vs normal AER	2	**2.5E-04**	7529
				Severe DKD	2	0.080	10000
				Any DKD	2	0.301	11223
				CKD	2	0.762	8269
				CKD extremes	2	0.764	7356
KIAA1109	Non-synonymous	SKAT	0.05	Any DKD	10	**3.0E-06**	11223
				Severe DKD	10	**5.7E-06**	10000
				CKD+DKD	10	**0.002**	6994
				CKD	10	**0.002**	8269
				Kidney failure vs normal AER	10	**0.011**	7529
				Severe albuminuria	10	**0.013**	7994
				CKD extremes	10	**0.015**	7356
				Kidney failure vs others	14	**0.023**	11996
				Moderate albuminuria	11	0.114	5840
				Kidney failure vs severe albuminuria	8	0.982	3273
LAIR1	Non-synonymous	SKAT	0.01	Kidney failure vs others	3	**1.3E-06**	11996
				Kidney failure vs normal AER	3	**8.7E-04**	7529
				CKD extremes	3	**0.038**	7356
				Severe DKD	3	0.148	10000
				CKD	3	0.153	8269
				Any DKD	3	0.285	11223
				CKD+DKD	2	0.768	6994
				Severe albuminuria	3	0.865	7994
				Moderate albuminuria	3	0.883	5840
MUC5B	Non-synonymous	burden	0.05	CKD	65	**6.7E-09**	8269
				CKD extremes	63	**1.6E-07**	7356
				Kidney failure vs others	68	**0.002**	11996
				Kidney failure vs normal AER	61	**0.010**	7529
				CKD+DKD	60	**0.017**	6994
				Kidney failure vs severe albuminuria	55	**0.032**	3273
				Severe albuminuria	63	0.201	7994
				Moderate albuminuria	61	0.718	5840

				Any DKD	64	0.799	11223
				Severe DKD	62	0.934	10000
PLCB2	Non-synonymous	burden	0.005	Kidney failure vs others	4	**2.2E-06**	11996
				Kidney failure vs normal AER	2	**0.002**	7529
				CKD extremes	2	**0.010**	7356
				CKD	2	**0.011**	8269
				Severe DKD	2	**0.026**	10000
				CKD+DKD	2	**0.040**	6994
				Any DKD	2	0.137	11223
				Moderate albuminuria	2	0.835	5840
ZAN	PTV	VT	0.019	CKD extremes	3	**3.4E-06**	7356
			0.021	Kidney failure vs severe albuminuria	3	**2.7E-05**	3273
			0.018	Kidney failure vs others	3	**8.2E-05**	11996
			0.020	Kidney failure vs normal AER	3	**0.003**	7529
			0.008	CKD	2	**0.003**	8269
			0.020	CKD+DKD	3	**0.012**	6994
			0.017	Severe albuminuria	3	0.124	7994
			0.037	Moderate albuminuria	5	0.684	5840
			0.019	Any DKD	3	0.851	11223

PTV: protein-truncating variants

Supplementary Table S13: Gene aggregate results ($p<3.4\times10^{-6}$ for non-synonymous variants, $p<2.5\times10^{-5}$ for protein-truncating variants) when also singletons and doubletons (i.e., minor allele count of 1 or 2) are included.

Results were filtered to genes with gene-wise cumulative minor allele count ≥ 5 across all studies and at least two variants found.

Gene	Phenotype	Var types	Freq	N var	Cum MAC	Test	*p*-value
IGSF3	Kidney failure vs others	Missense	0.5%, 1%	3	14	SKAT	2.07×10^{-10}
	Kidney failure vs others	Missense	0.14%	2	7	VT	3.47×10^{-8}
MUC5B	CKD	Missense	5%	72	13,214	burden	3.37×10^{-9}
	CKD	Missense	4.8%	70	11,604	VT	4.64×10^{-9}
	CKD	Missense	5%	72	13,214	SKAT	2.12×10^{-6}
	CKD extremes	Missense	5%	72	11,423	burden	2.27×10^{-8}
	CKD extremes	Missense	4.9%	70	9,965	VT	6.46×10^{-8}
LAIR1	Kidney failure vs others	Missense	0.5%, 1%	4	23	SKAT	1.15×10^{-6}
SETX	Severe albuminuria	Missense	0.5%	5	25	SKAT	1.47×10^{-6}
PLCB2	Kidney failure vs others	Missense	0.5%	5	51	burden	1.69×10^{-6}
ATP1A4	CKD	Missense, stop gain	0.5%	6	117	burden	1.99×10^{-6}
EOMES	Kidney failure vs others	Missense	5%, 1%	4	227	SKAT	2.32×10^{-6}
SIPA1	Moderate albuminuria	Missense	5%, 1%, 0.5%	3	49	SKAT	2.61×10^{-6}
EMILIN1	Kidney failure vs Ctrl	Missense, stop gain	0.5%	2	11	SKAT	2.99×10^{-6}
KIAA1109	Any DKD	Missense, splice donor	5%	17	2,431	SKAT	3.00×10^{-6}
	Severe DKD	Missense, splice donor	5%	17	2,192	SKAT	3.17×10^{-6}
ZAN	CKD extremes	splice donor, frameshift, stop gained	1.9%	4	324	VT	4.86×10^{-6}

Note, only variants with MAC≥3 were included in the original gene aggregate analyses. Var types: variant types. Freq: variant frequency threshold. N var, number of variants of the given variant type and below the given variant frequency threshold found within the gene. Cum MAC, cumulative minor allele count across all variants within the gene, across all studies. Test, gene aggregate test; SKAT, sequence kernel association test; VT, variable threshold burden test.

Supplementary Table S14: Physicians and nurses contributing to the patient recruitment and examination

FinnDiane Study Centers	Physicians and nurses
Anjalankoski Health Centre	S. Koivula, T. Uggeldahl
Central Finland Central Hospital, Jyväskylä	T. Forslund, A. Halonen, A. Koistinen, P. Koskiaho, M. Laukkanen, J. Saltevo, M. Tiihonen
Central Hospital of Åland Islands, Mariehamn	M. Forsen, H. Granlund, A-C. Jonsson, B. Nyroos
Central Hospital of Kanta-Häme, Hämeenlinna	P. Kinnunen, A. Orvola, T. Salonen, A. Vähänen
Central Hospital of Länsi-Pohja, Kemi	H. Laukkanen, P. Nyländen, A. Sademies
Central Ostrabothnian Hospital District, Kokkola	S. Anderson, B. Asplund, U. Byskata, P. Liedes, M. Kuusela, T. Virkkala
City of Espoo Health Centre	
Espoonlahti	A. Nikkola, E. Ritola
Tapiola	M. Niska, H. Saarinen
Samaria	E. Oukko-Ruponen, T. Virtanen
Viherlaakso	A. Lyytinen
City of Helsinki Health Centre	
Puistola	H. Kari, T. Simonen
Suutarila	A. Kaprio, J. Kärkkäinen, B. Rantaeskola
Töölö	P. Kääriäinen, J. Haaga, A-L. Pietiläinen
City of Hyvinkää Health Centre	S. Klemetti, T. Nyandoto, E. Rontu, S. Satuli-Autere
City of Vantaa Health Centre	
Korso	R. Toivonen, H. Virtanen
Länsimäki	R. Ahonen, M. Ivaska-Suomela, A. Jauhiainen
Martinlaakso	M. Laine, T. Pellonpää, R. Puranen
Myyrmäki	A. Airas, J. Laakso, K. Rautavaara
Rekola	M. Erola, E. Jatkola
Tikkurila	R. Lönnblad, A. Malm, J. Mäkelä, E. Rautamo
Heinola Health Centre	P. Hentunen, J. Lagerstam
Helsinki University Central Hospital, Department of Medicine, Division of Nephrology	A. Ahola, J. Fagerudd, M. Feodoroff, D. Gordin, O. Heikkilä, K Hietala, L. Kyllönen, J. Kytö, S. Lindh, K. Pettersson-Fernholm, M. Rosengård-Bärlund, M. Rönnback, A. Sandelin, A-R Salonen, L. Salovaara, L. Thorn, J. Tuomikangas, T. Vesisenaho, J. Wadén
Herttoniemi Hospital, Helsinki	V. Sipilä
Hospital of Lounais-Häme, Forssa	T. Kalliomäki, J. Koskelainen, R. Nikkanen, N. Savolainen, H. Sulonen, E. Valtonen
Iisalmi Hospital	E. Toivanen
Jokilaakso Hospital, Jämsä	A. Parta, I. Pirttiniemi
Jorvi Hospital, Helsinki University Central Hospital	S. Aranko, S. Ervasti, R. Kauppinen-Mäkelin, A. Kuusisto, T. Leppälä, K. Nikkilä, L. Pekkonen
Jyväskylä Health Centre, Kyllö	K. Nuorva, M. Tiihonen
Kainuu Central Hospital, Kajaani	S. Jokelainen, P. Kemppainen, A-M. Mankinen, M. Sankari
Kerava Health Centre	H. Stuckey, P. Suominen

FinnDiane Study Centers	Physicians and nurses
Kirkkonummi Health Centre	A. Lappalainen, M. Liimatainen, J. Santaholma
Kivelä Hospital, Helsinki	A. Aimolahti, E. Huovinen
Koskela Hospital, Helsinki	V. Ilkka, M. Lehtimäki
Kotka Heath Centre	E. Pälikkö-Kontinen, A. Vanhanen
Kouvola Health Centre	E. Koskinen, T. Siitonen
Kuopio University Hospital	E. Huttunen, R. Ikäheimo, P. Karhapää, P. Kekäläinen, M. Laakso, T. Lakka, E. Lampainen, L. Moilanen, L. Niskanen, U. Tuovinen, I. Vauhkonen, E. Voutilainen
Kuusamo Health Centre	T. Kääriäinen, E. Isopoussu
Kuusankoski Hospital	E. Kilkki, I. Koskinen, L. Riihelä
Laakso Hospital, Helsinki	T. Meriläinen, P. Poukka, R. Savolainen, N. Uhlenius
Lahti City Hospital	A. Mäkelä, M. Tanner
Lapland Central Hospital, Rovaniemi	L. Hyvärinen, S. Severinkangas, T. Tulokas
Lappeenranta Health Centre	P. Linkola, I. Pulli
Lohja Hospital	T. Granlund, M. Saari, T. Salonen
Loimaa Health Centre	A. Mäkelä, P. Eloranta
Länsi-Uusimaa Hospital, Tammisaari	I-M. Jousmaa, J. Rinne
Malmi Hospital, Helsinki	H. Lanki, S. Moilanen, M. Tilly-Kiesi
Mikkeli Central Hospital	A. Gynther, R. Manninen, P. Nironen, M. Salminen, T. Vänttinen
Mänttä Regional Hospital	I. Pirttiniemi, A-M. Hänninen
North Karelian Hospital, Joensuu	U-M. Henttula, P. Kekäläinen, M. Pietarinen, A. Rissanen, M. Voutilainen
Nurmijärvi Health Centre	A. Burgos, K. Urtamo
Oulankangas Hospital, Oulainen	E. Jokelainen, P-L. Jylkkä, E. Kaarlela, J. Vuolaspuro
Oulu Health Centre	L. Hiltunen, R. Häkkinen, S. Keinänen-Kiukaanniemi
Oulu University Hospital	R. Ikäheimo
Päijät-Häme Central Hospital	H. Haapamäki, A. Helanterä, S. Hämäläinen, V. Ilvesmäki, H. Miettinen
Palokka Health Centre	P. Sopanen, L. Welling
Pieksämäki Hospital	V. Javtsenko, M. Tamminen
Pietarsaari Hospital	M-L. Holmbäck, B. Isomaa, L. Sarelin
Pori City Hospital	P. Ahonen, P. Merensalo, K. Sävelä
Porvoo Hospital	M. Kallio, B. Rask, S. Rämö
Raahe Hospital	A. Holma, M. Honkala, A. Tuomivaara, R. Vainionpää
Rauma Hospital	K. Laine, K. Saarinen, T. Salminen
Riihimäki Hospital	P. Aalto, E. Immonen, L. Juurinen
Salo Hospital	A. Alanko, J. Lapinleimu, P. Rautio, M. Virtanen
Satakunta Central Hospital, Pori	M. Asola, M. Juhola, P. Kunelius, M-L. Lahdenmäki, P. Pääkkönen, M. Rautavirta
Savonlinna Central Hospital	E. Korpi-Hyövälti, T. Latvala, E. Leijala
South Karelia Central Hospital, Lappeenranta	T. Ensala, E. Hussi, R. Härkönen, U. Nyholm, J. Toivanen
Tampere Health Centre	A. Vaden, P. Alarotu, E. Kujansuu, H. Kirkkopelto-Jokinen, M. Helin, S. Gummerus, L. Calonius, T. Niskanen, T. Kaitala, T. Vatanen

FinnDiane Study Centers	Physicians and nurses
Tampere University Hospital	I. Ala-Houhala, T. Kuningas, P. Lampinen, M. Määttä, H. Oksala, T. Oksanen, K. Salonen, H. Tauriainen, S. Tulokas
Tiirismaa Health Centre, Hollola	T. Kivelä, L, Petlin, L. Savolainen
Turku Health Centre	I. Hämäläinen, H. Virtamo, M. Vähätalo
Turku University Central Hospital	K. Breitholz, R. Eskola, K. Metsärinne, U. Pietilä, P. Saarinen, R. Tuominen, S. Äyräpää
Vaajakoski Health Centre	K. Mäkinen, P. Sopanen
Valkeakoski Regional Hospital	S. Ojanen, E. Valtonen, H. Ylönen, M. Rautiainen, T. Immonen
Vammala Regional Hospital	I. Isomäki, R. Kroneld, M. Tapiolinna-Mäkelä
Vaasa Central Hospital	S. Bergkulla, U. Hautamäki, V-A. Myllyniemi, I. Rusk

RomDiane - Study Centres	Physicians, students and nurses
”N.C Paulescu” National Institute for DiabetesNutrition and Metabolic Diseases from Bucharest	Anghel M, Cheta DM, Cimpoca BA, Cozma CN, Dandu N, Dobrin D, Duta I, Frentescu AM, Ionescu-Tirgoviste C, Ionica R, Lichiardopol R, Oprea AL, Panduru NM, Pop A, Pop G, Radescu R, Radu S, Robu M, Serafinceanu C, Tudose O
Craiova Emergency County Hospital	Bacu M, Clenciu D, Graunteanu C, Mota E, Mota M

LatDiane acknowledges the following doctors and researchers	
	A. Bogdanova, D. Grikmane, I. Care, A. Fjodorova, R. Graudina, A. Valtere, D. Teterovska, I. Dzivite-Krišane, I. Kirilova, U. Lauga-Turina, K. Geldnere, D. Seisuma, N. Fokina, S. Steina, L. Jaunozola, I. Balcere, U. Gailiša, E. Menise, S. Broka, L. Akmene, N. Kapļa, J. Nagaiceva, A. Petersons, A. Lejnieks, I. Konrade, I. Salna, A. Dekante, A. Gramatniece, A. Salina, A. Silda, V.Mešečko, V.Mihejeva, K. Kudrjavceva, I. Marksa, M. Cirse, D. Zeme, S. Kalva-Vaivode,J. Klovinš, L. Nikitina-Zake, V. Rovite, S. Skrebinska, Z. Dzerve, and R.Mallons.

Supplementary Figure S1: Power calculation for Severe DKD phenotype (N=6,177 controls with normal AER, N=3,823 cases with severe albuminuria or kidney failure), for α=5×10^{-7} significance level.

Power calculated for minor allele frequencies (MAF) 0.5%, 1%, 2%, 3%, 4%, 5%, and 10% with R genpwr package.

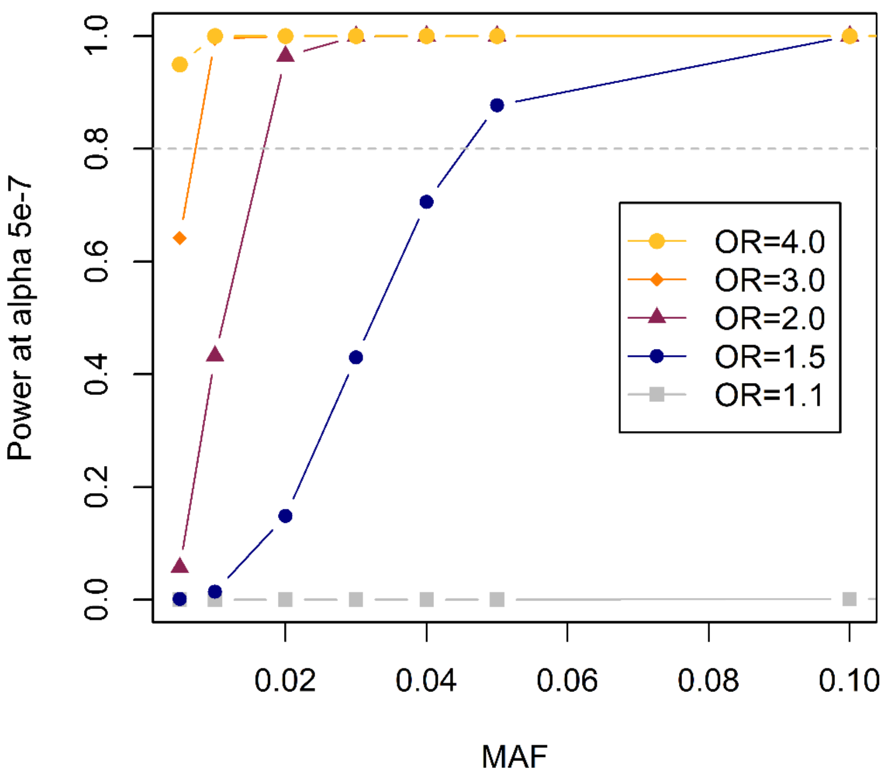

Supplementary Figure S2: Single variant analysis Manhattan and QQ-plots for each tested phenotype. Plotted with topR R package.[S16]

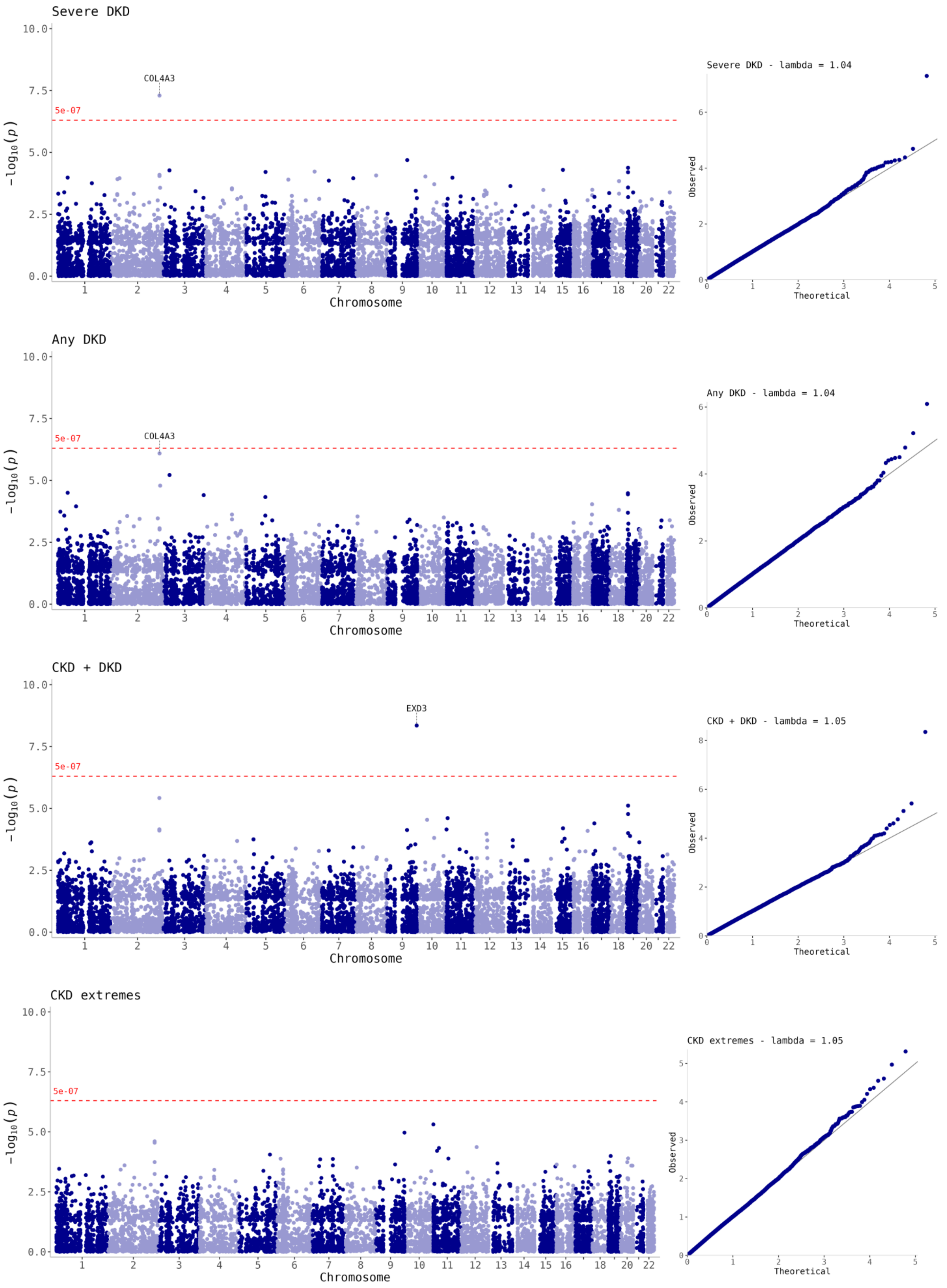

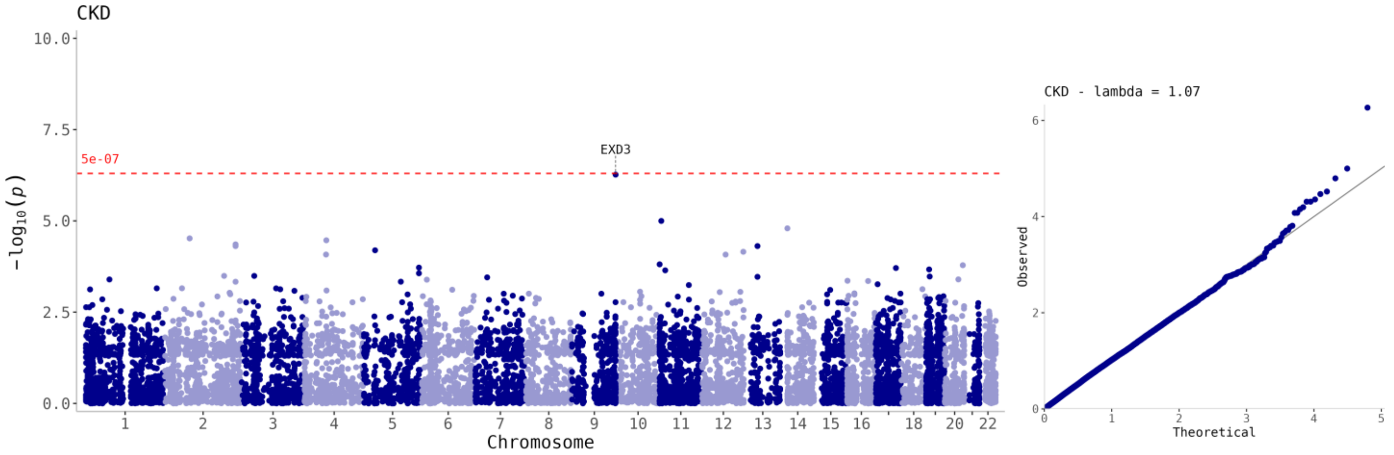
CKD
EXD3
5e-07
−log10(p)
Chromosome
CKD - lambda = 1.07
Observed
Theoretical

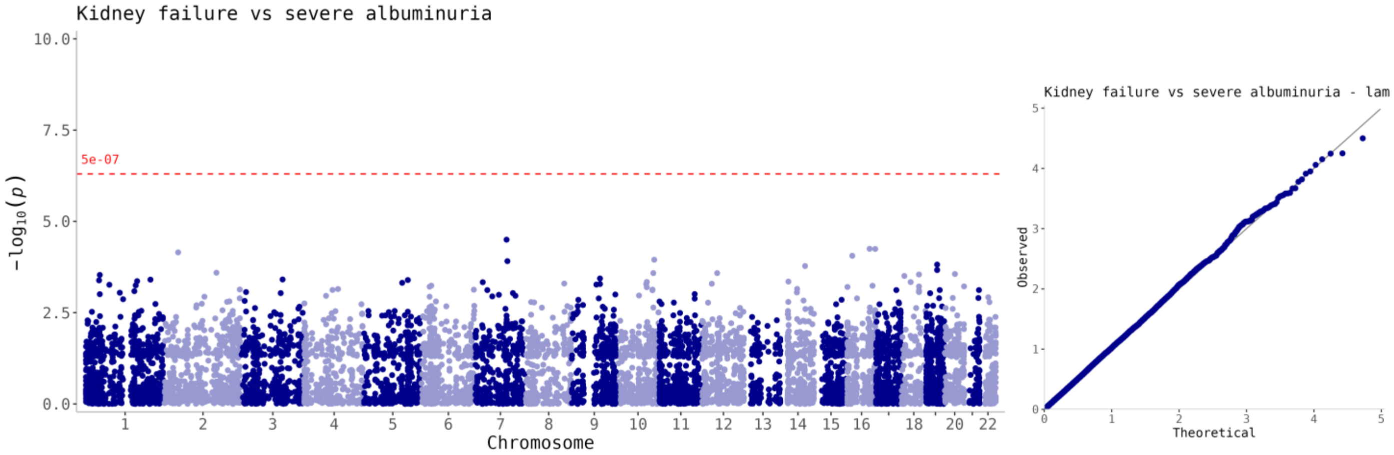
Kidney failure vs severe albuminuria
5e-07
−log10(p)
Chromosome
Kidney failure vs severe albuminuria - lam
Observed
Theoretical

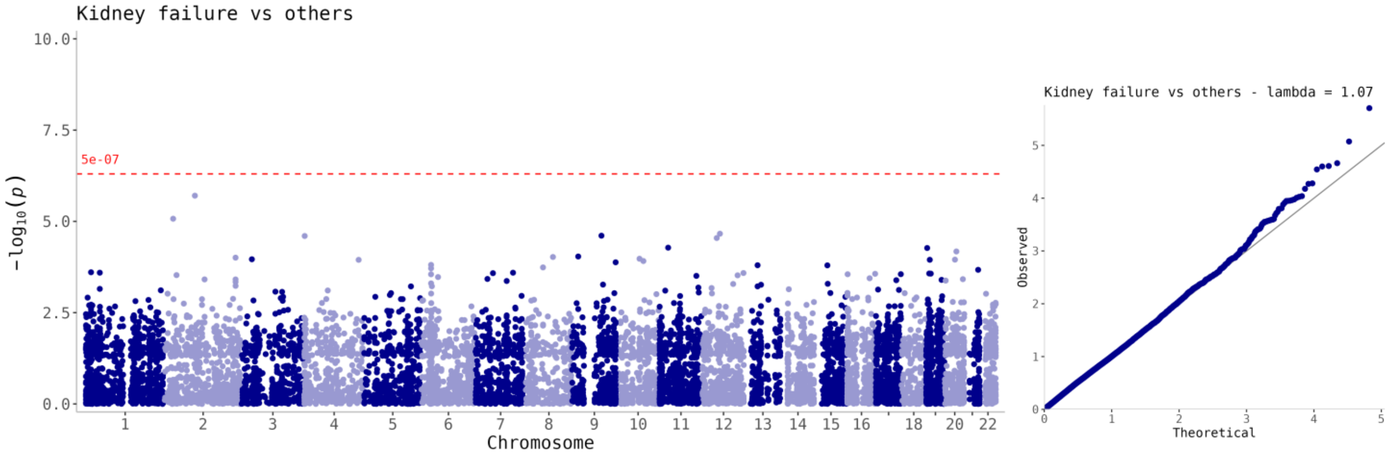
Kidney failure vs others
5e-07
−log10(p)
Chromosome
Kidney failure vs others - lambda = 1.07
Observed
Theoretical

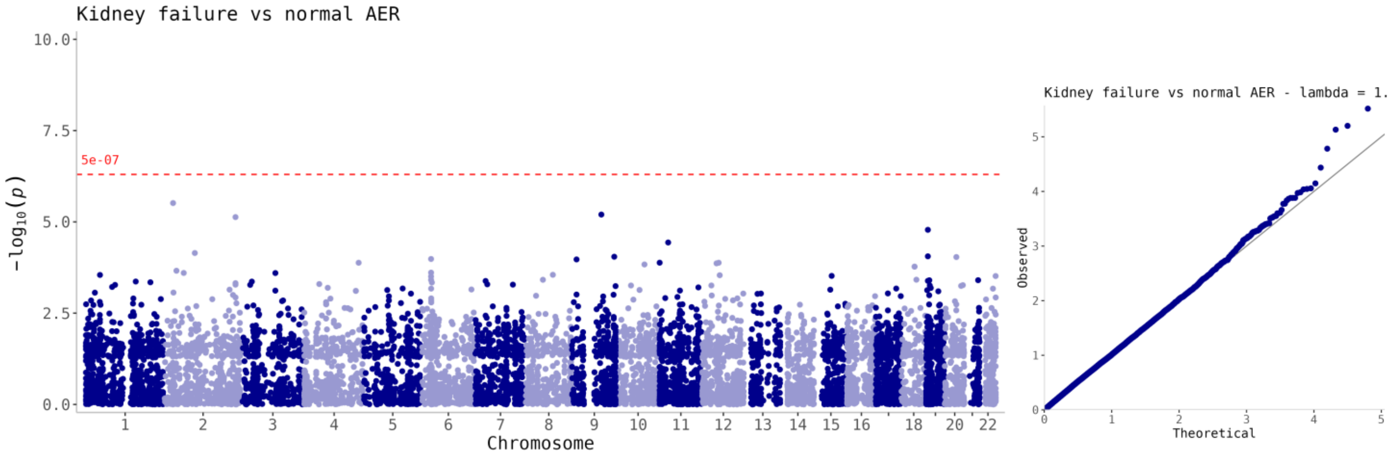
Kidney failure vs normal AER
5e-07
−log10(p)
Chromosome
Kidney failure vs normal AER - lambda = 1.
Observed
Theoretical

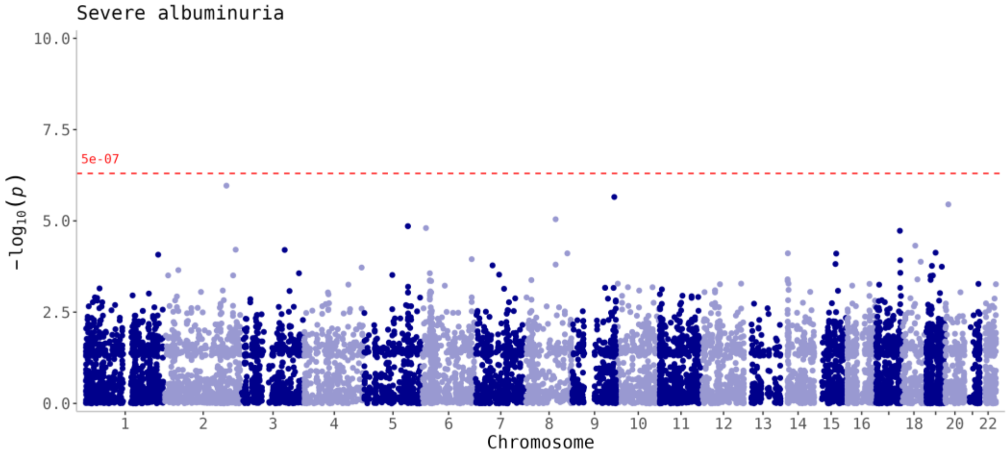
Severe albuminuria
5e-07
−log10(p)
Chromosome

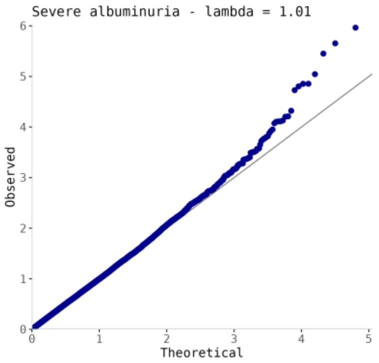
Severe albuminuria - lambda = 1.01
Observed
Theoretical

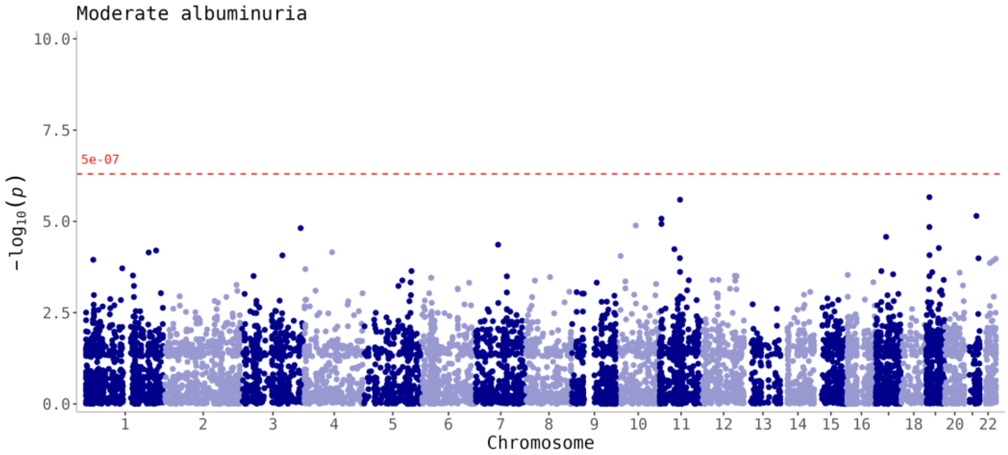
Moderate albuminuria
5e-07
−log10(p)
Chromosome

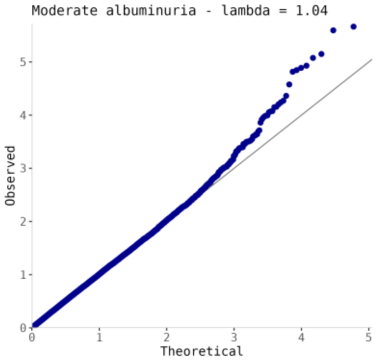
Moderate albuminuria - lambda = 1.04
Observed
Theoretical

Supplementary Figure S3: *EXD3* p.Asp555Asn variant (rs200080727) association with CKD+DKD ($p=4.5\times10^{-9}$).

A: Regional association plot, plotted with topR R package.[S16] **B:** *EXD3* has multiple isoforms. The exon including *EXD3* p.Asp555Asn (rs200080727) is indicated with red box, and present in four transcripts of which three are expressed in kidney cortex. **C:** These transcripts are expressed in kidney cortex and medulla among other tissues (data from GTExPortal (gtexportal.org) on 04/30/2024).

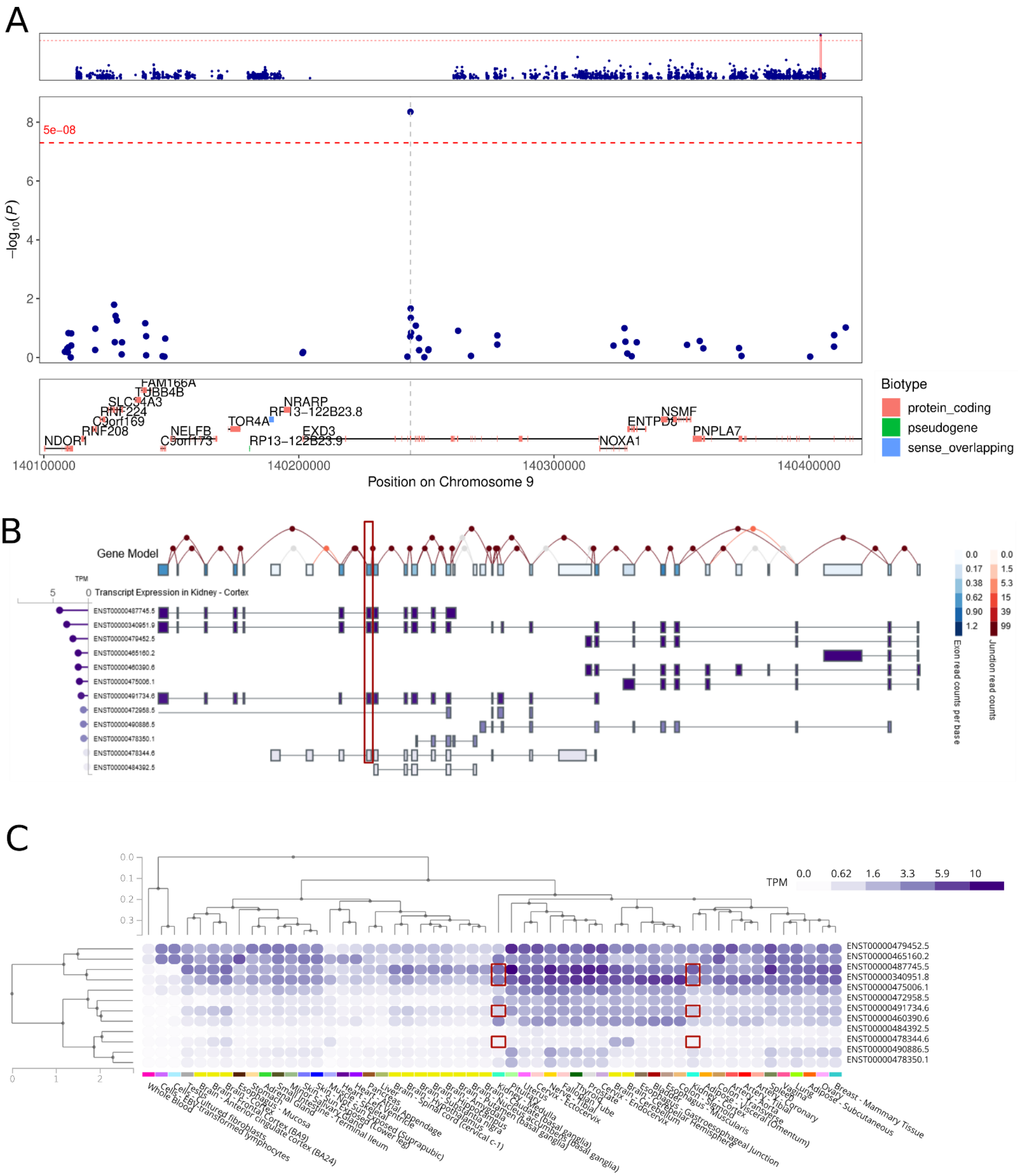

Supplementary Figure S4: *EXD3* and *MUC5B* gene expression in single nucleus RNA sequencing (snRNAseq) datasets. Data queried from http://humphreyslab.com/SingleCell/ [S17]

Healthy Human adult kidney – Complete: 4,524 nuclei

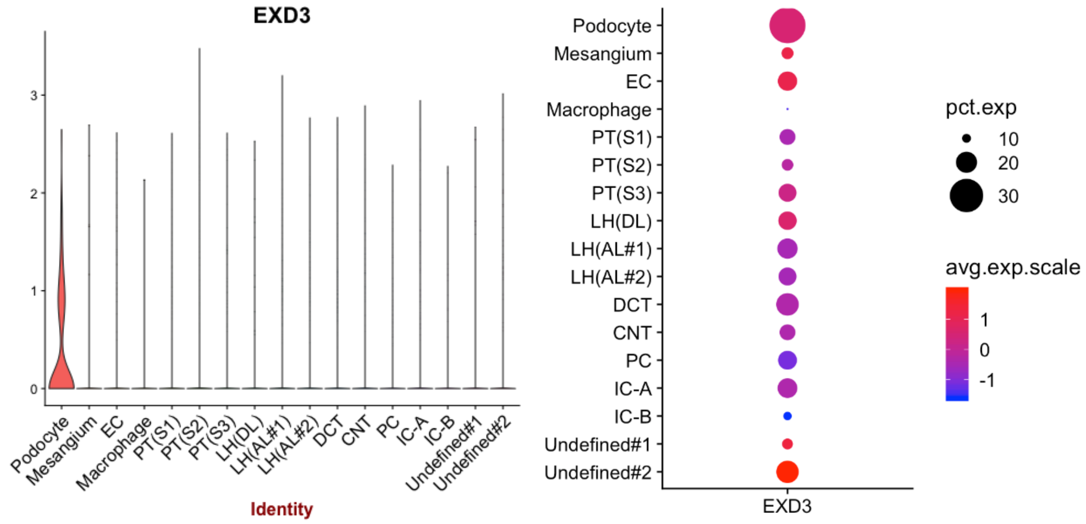

Human Diabetic Kidney (Wilson et al., 2019): 23,980 nuclei[S18]

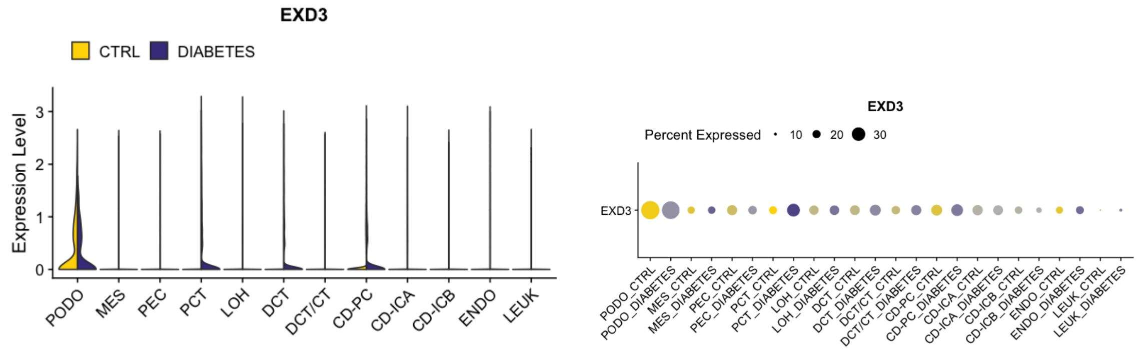

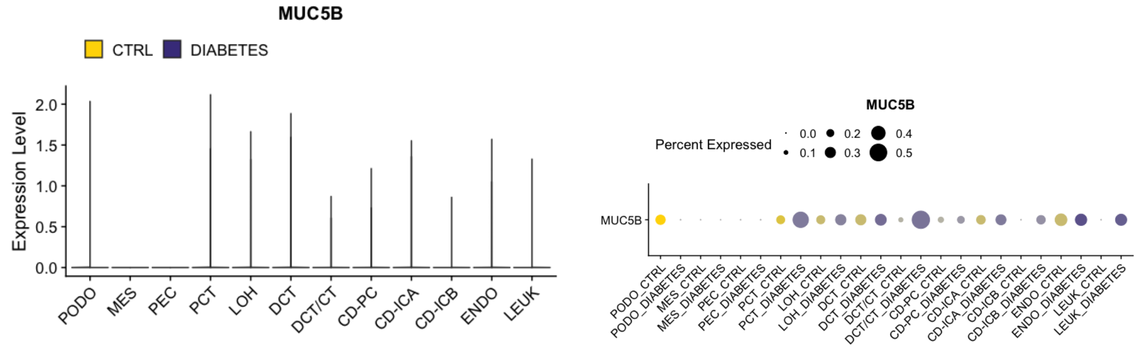

Supplementary Figure S5: Manhattan and QQ-plots for the gene aggregate tests. Left panels: Manhattan plots, with gene aggregate test results for non-synonymous variants are drawn with purple color, significant associations defined as p-values <3.4×10-6 (upper dashed red line, corrected for 14,963 genes with non-synonymous variants). Results for protein-truncating variants (PTV) are drawn with orange color, significant associations defined as p-values <2.5×10-5 (lower dashed red line, corrected for 2,013 genes with protein-truncating variants). Middle panels: Quantile-Quantile plot (QQ-plot) for gene aggregate tests of non-synonymous variants, stratified by aggregate test (burden, SKAT, or variable threshold (VT)) and variant frequency (0.05, 0.01 or 0.005). Right panels: QQ-plot for gene aggregate tests of protein-truncating variants, stratified by aggregate test and variant frequency.

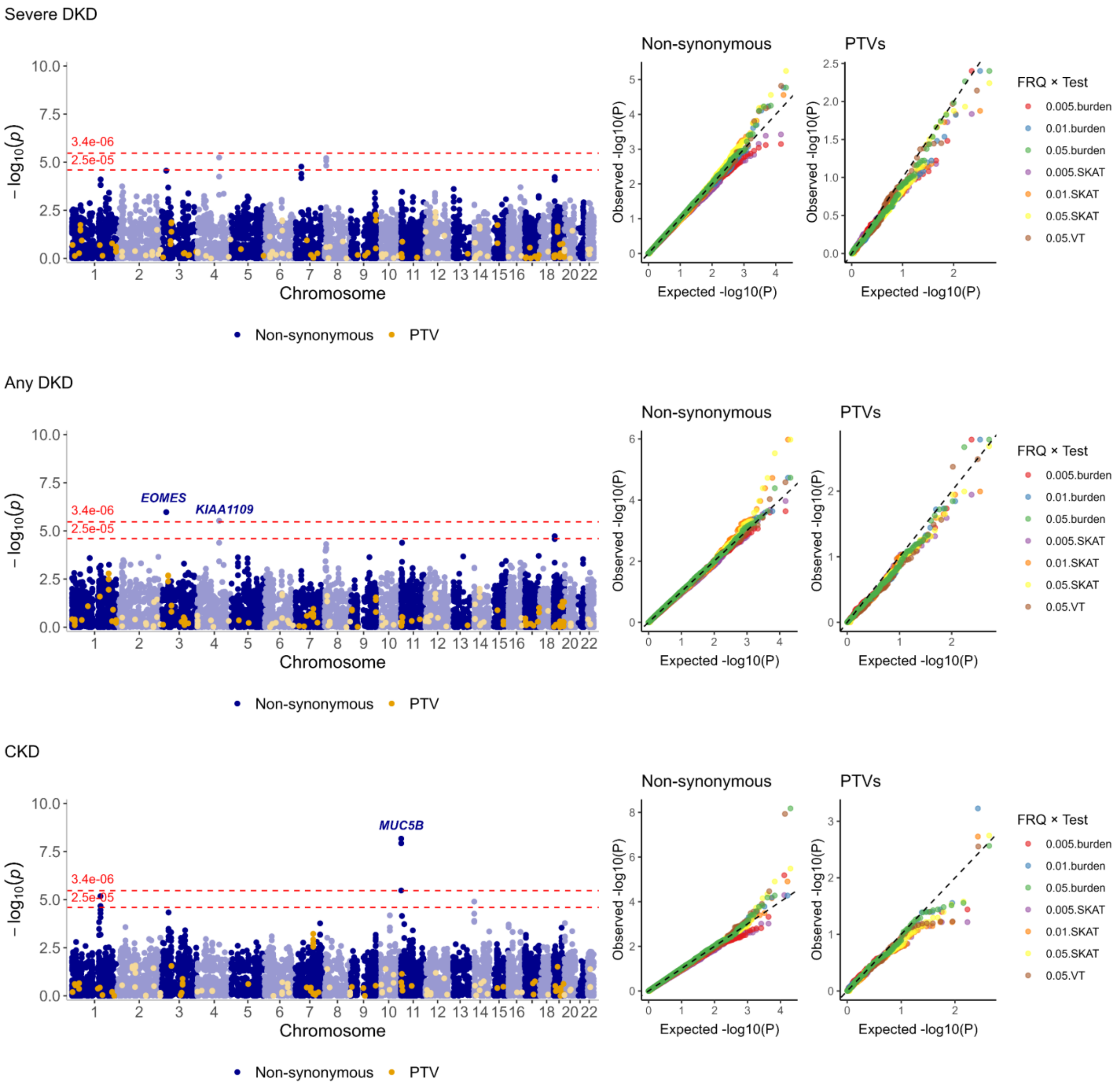

CKD + DKD

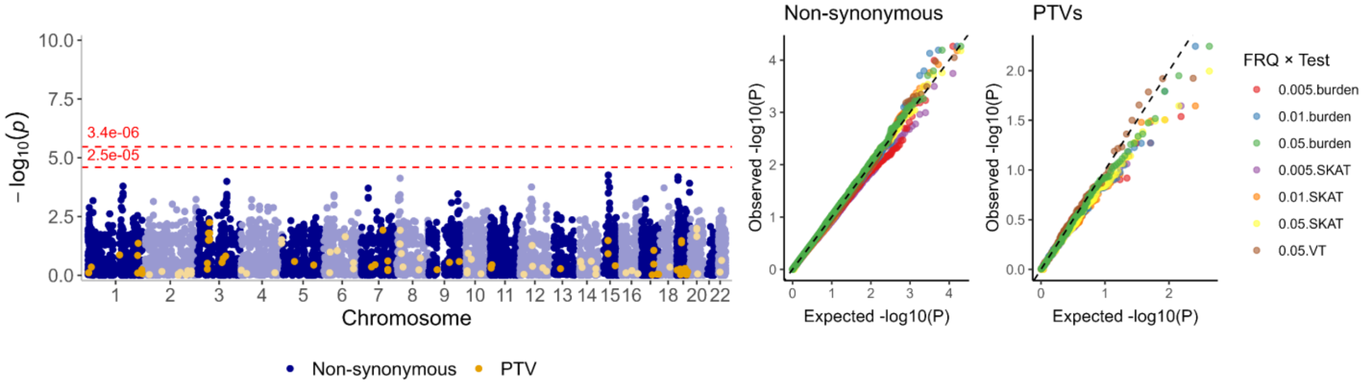

CKD extremes

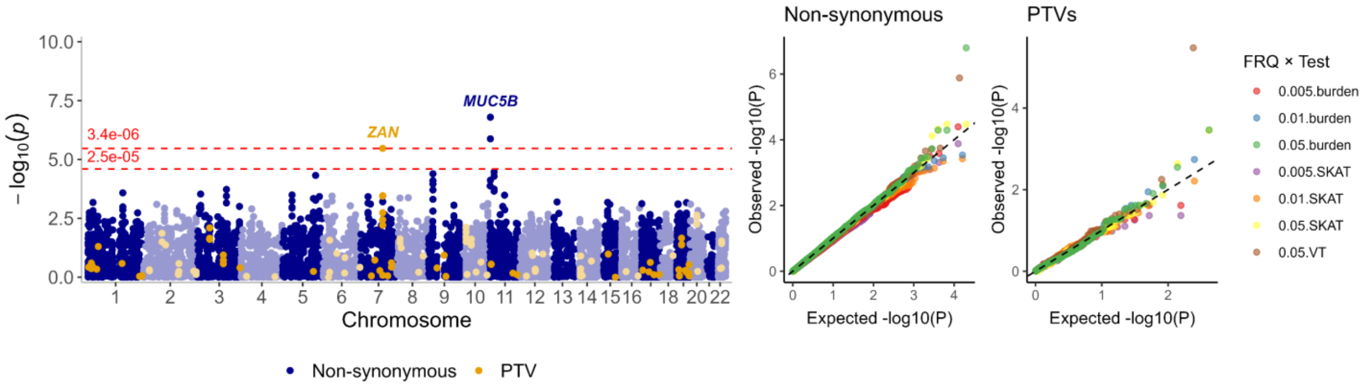

Kidney failure vs others

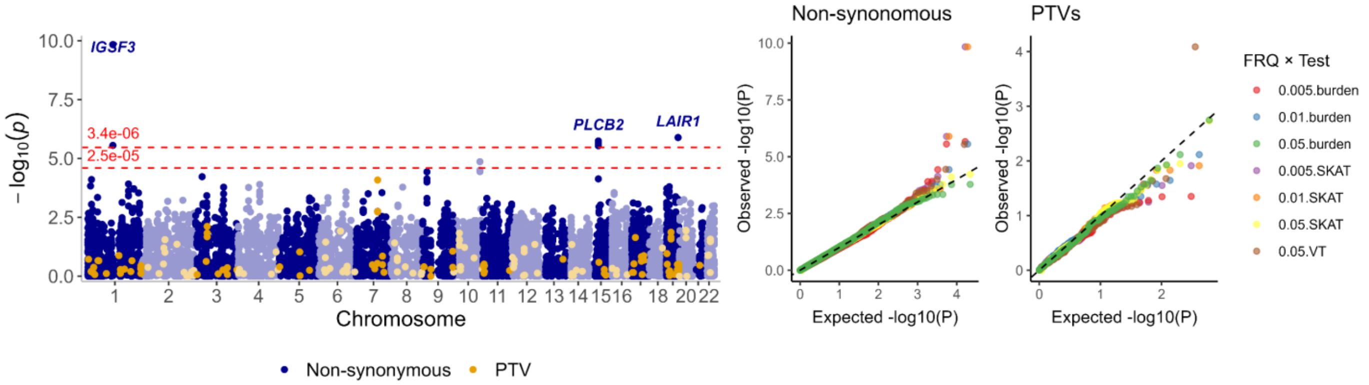

Kidney failure vs normal AER

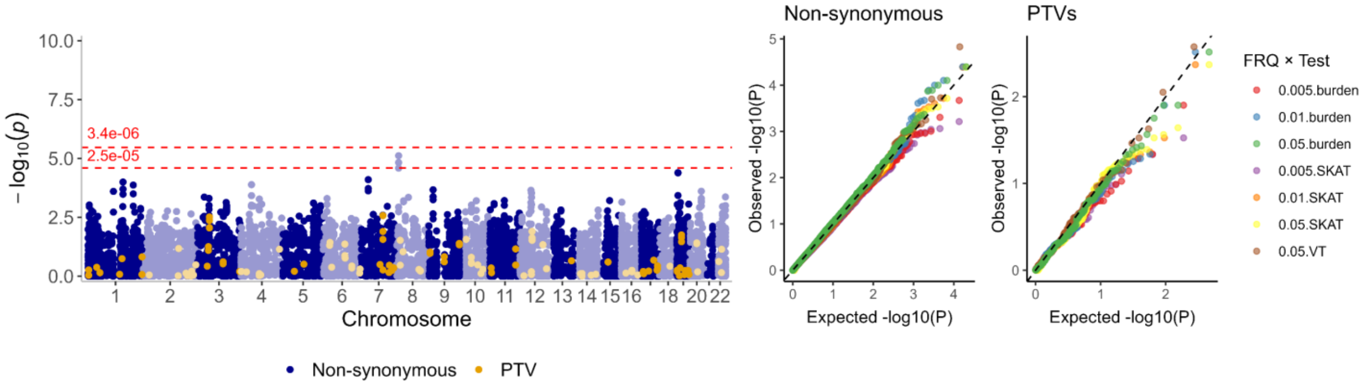

Kidney failure vs severe albuminuria

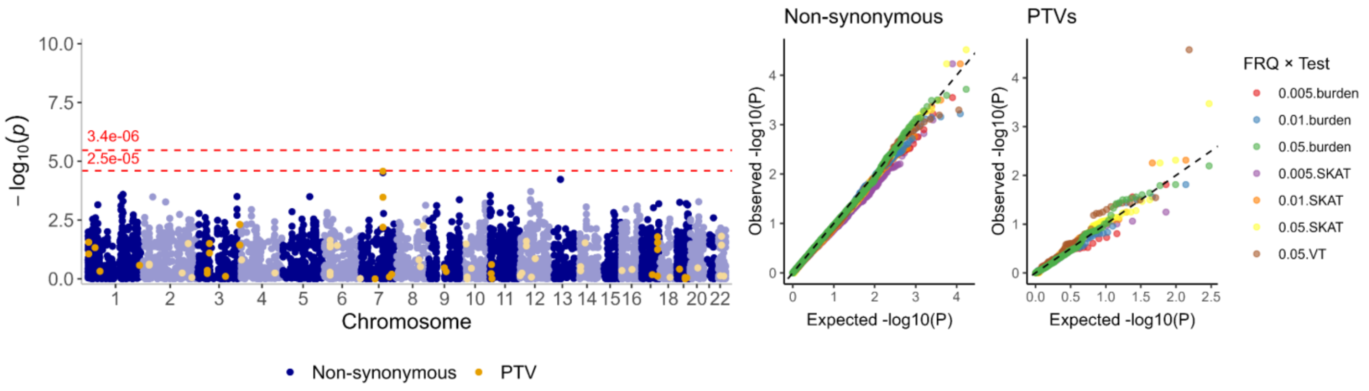

Severe albuminuria

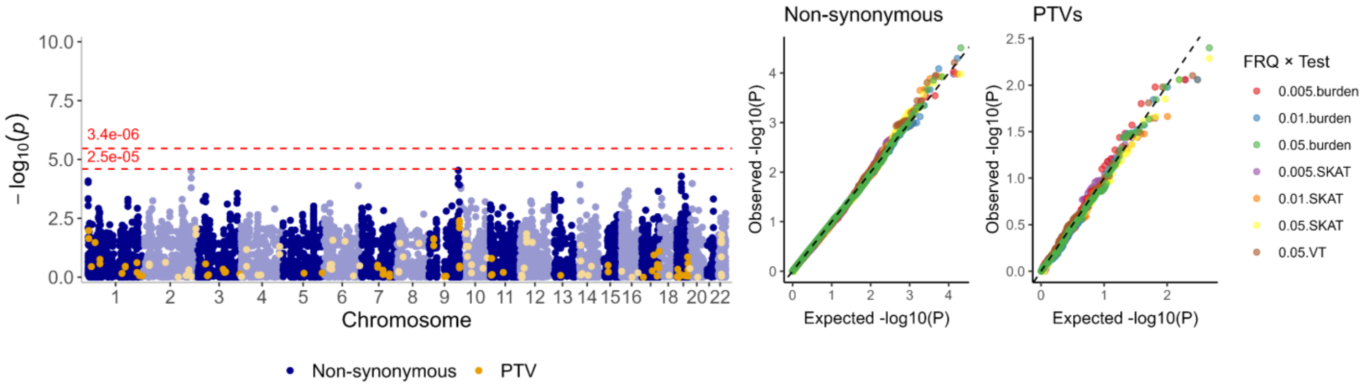

Moderate albuminuria

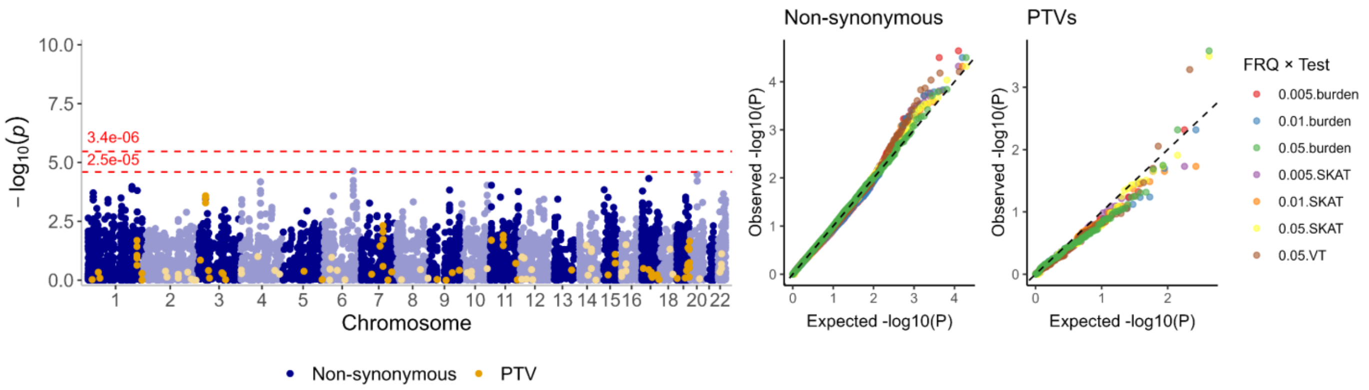

Supplementary Figure S6: Linkage disequilibrium (LD) structure between the *MUC5B* missense variants, the *MUC5B* promoter variant associated with gene expression (rs35705950), and rs2672810 tagging for a *MUC5B* VNTR[S19]**. A:** All PAVs detected in discovery stage. Variants with *P*<0.05 highlighted in yellow. **B:** PAVs with *P*<0.05 in discovery stage, together with the *MUC5B* promoter variant rs35705950 and rs2672810 tagging VNTR length variation in the *MUC5B* large central exon (r^2=0.66)[S19].

A **B**

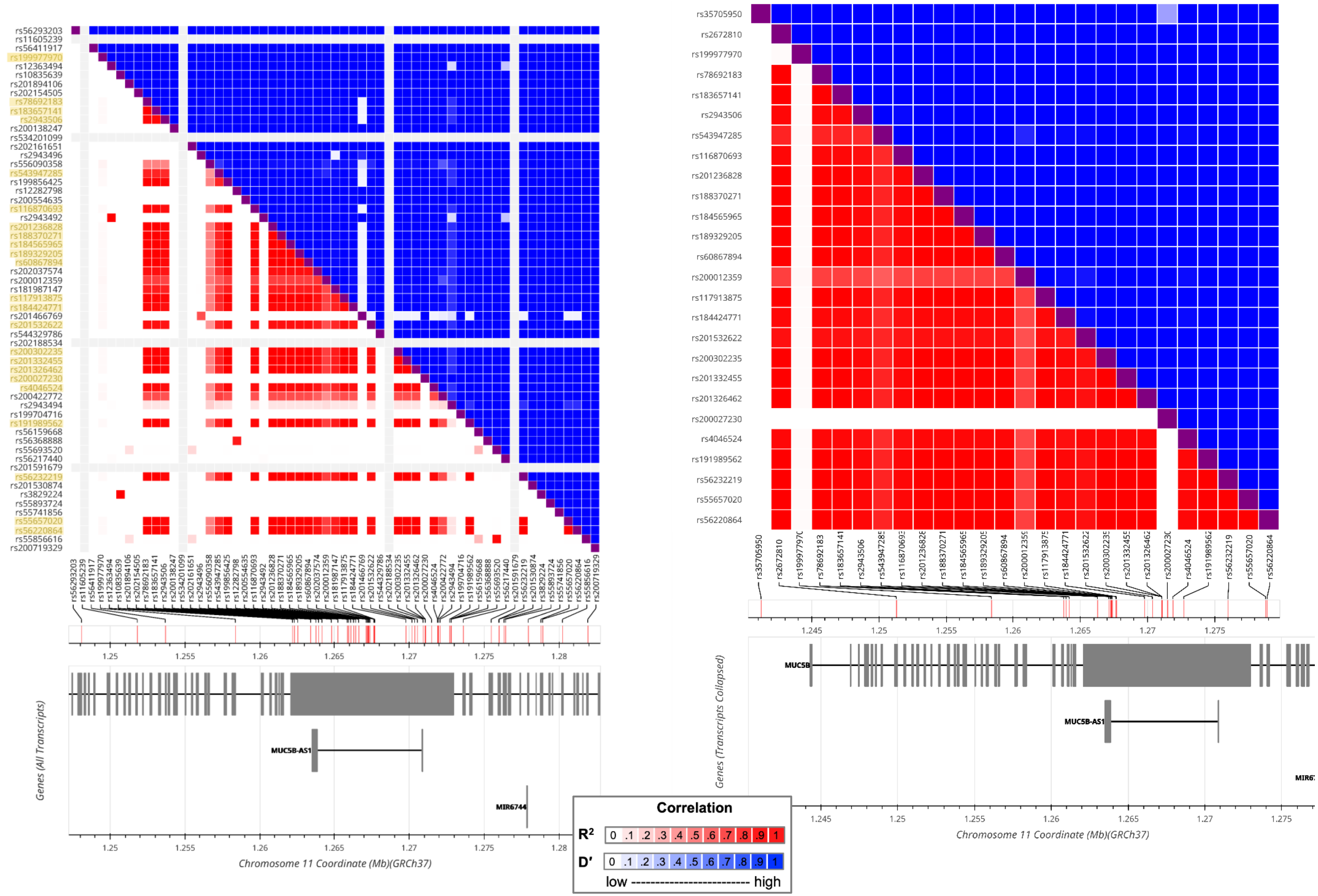

Supplementary References

S1. Salem RM, Todd JN, Sandholm N, Cole JB, Chen W-M, Andrews D, et al.: Genome-Wide Association Study of Diabetic Kidney Disease Highlights Biology Involved in Glomerular Basement Membrane Collagen. *J Am Soc Nephrol* 30: 2000–2016, 2019

S2. Sandholm N, Hotakainen R, Haukka JK, Jansson Sigfrids F, Dahlström EH, Antikainen AA, et al.: Whole-exome sequencing identifies novel protein-altering variants associated with serum apolipoprotein and lipid concentrations. *Genome Med* 14: 132, 2022

S3. Kircher M, Witten DM, Jain P, O'Roak BJ, Cooper GM, Shendure J: A general framework for estimating the relative pathogenicity of human genetic variants. *Nat Genet* 46: 310–315, 2014

S4. Tordai H, Torres O, Csepi M, Padányi R, Lukács GL, Hegedűs T: Analysis of AlphaMissense data in different protein groups and structural context. *Sci Data* 11: 495, 2024

S5. Eastwood SV, Mathur R, Atkinson M, Brophy S, Sudlow C, Flaig R, et al.: Algorithms for the Capture and Adjudication of Prevalent and Incident Diabetes in UK Biobank. *PLoS One* 11: e0162388, 2016

S6. Pattaro C, Riegler P, Stifter G, Modenese M, Minelli C, Pramstaller PP: Estimating the glomerular filtration rate in the general population using different equations: effects on classification and association. *Nephron Clin Pract* 123: 102–111, 2013

S7. Haukka JK, Antikainen AA, Valo E, Syreeni A, Dahlström EH, Lin BM, et al.: Whole-exome and whole-genome sequencing of 1064 individuals with type 1 diabetes reveals novel genes for diabetic kidney disease. *Diabetologia* 67: 2494–2506, 2024

S8. Nakagawa S, Nishihara K, Miyata H, Shinke H, Tomita E, Kajiwara M, et al.: Molecular Markers of Tubulointerstitial Fibrosis and Tubular Cell Damage in Patients with Chronic Kidney Disease. *PLoS One* 10: e0136994, 2015

S9. Woroniecka KI, Park AS, Mohtat D, Thomas DB, Pullman JM, Susztak K: Transcriptome analysis of human diabetic kidney disease. *Diabetes* 60: 2354–2369, 2011

S10. Levin A, Reznichenko A, Witasp A, Liu P, Greasley PJ, Sorrentino A, et al.: Novel insights into the disease transcriptome of human diabetic glomeruli and tubulointerstitium. *Nephrol Dial Transplant* 35: 2059–2072, 2020

S11. Fan Y, Yi Z, D'Agati VD, Sun Z, Zhong F, Zhang W, et al.: Comparison of Kidney Transcriptomic Profiles of Early and Advanced Diabetic Nephropathy Reveals Potential New Mechanisms for Disease Progression. *Diabetes* 68: 2301–2314, 2019

S12. Schmid H, Boucherot A, Yasuda Y, Henger A, Brunner B, Eichinger F, et al.: Modular activation of nuclear factor-kappaB transcriptional programs in human diabetic nephropathy. *Diabetes* 55: 2993–3003, 2006

S13. Ju W, Greene CS, Eichinger F, Nair V, Hodgin JB, Bitzer M, et al.: Defining cell-type specificity at the transcriptional level in human disease. *Genome Res* 23: 1862–1873, 2013

S14. Hill C, Duffy S, Kettyle LM, McGlynn L, Sandholm N, Salem RM, et al.: Differential Methylation of Telomere-Related Genes Is Associated with Kidney Disease in Individuals with Type 1 Diabetes. *Genes (Basel)* 14: 1029, 2023

S15. Abedini A, Levinsohn J, Klötzer KA, Dumoulin B, Ma Z, Frederick J, et al.: Single-cell multi-omic and spatial profiling of human kidneys implicates the fibrotic microenvironment in kidney disease progression. *Nat Genet* 56: 1712–1724, 2024

S16. Juliusdottir T: topr: an R package for viewing and annotating genetic association results. *BMC Bioinformatics* 24: 268, 2023

S17. Wu H, Malone AF, Donnelly EL, Kirita Y, Uchimura K, Ramakrishnan SM, et al.: Single-Cell Transcriptomics of a Human Kidney Allograft Biopsy Specimen Defines a Diverse Inflammatory Response. *J Am Soc Nephrol* 29: 2069–2080, 2018

S18. Wilson PC, Wu H, Kirita Y, Uchimura K, Ledru N, Rennke HG, et al.: The single-cell transcriptomic landscape of early human diabetic nephropathy. *Proc Natl Acad Sci U S A* 116: 19619–19625, 2019

S19. Mukamel RE, Handsaker RE, Sherman MA, Barton AR, Hujoel MLA, McCarroll SA, et al.: Repeat polymorphisms underlie top genetic risk loci for glaucoma and colorectal cancer. *Cell* 186: 3659-3673.e23, 2023
